# Gestational weight gain charts for different body mass index groups for women in Europe, North America, and Oceania

**DOI:** 10.1186/s12916-018-1189-1

**Published:** 2018-11-05

**Authors:** Susana Santos, Iris Eekhout, Ellis Voerman, Romy Gaillard, Henrique Barros, Marie-Aline Charles, Leda Chatzi, Cécile Chevrier, George P. Chrousos, Eva Corpeleijn, Nathalie Costet, Sarah Crozier, Myriam Doyon, Merete Eggesbø, Maria Pia Fantini, Sara Farchi, Francesco Forastiere, Luigi Gagliardi, Vagelis Georgiu, Keith M. Godfrey, Davide Gori, Veit Grote, Wojciech Hanke, Irva Hertz-Picciotto, Barbara Heude, Marie-France Hivert, Daniel Hryhorczuk, Rae-Chi Huang, Hazel Inskip, Todd A. Jusko, Anne M. Karvonen, Berthold Koletzko, Leanne K. Küpers, Hanna Lagström, Debbie A. Lawlor, Irina Lehmann, Maria-Jose Lopez-Espinosa, Per Magnus, Renata Majewska, Johanna Mäkelä, Yannis Manios, Sheila W. McDonald, Monique Mommers, Camilla S. Morgen, George Moschonis, Ľubica Murínová, John Newnham, Ellen A. Nohr, Anne-Marie Nybo Andersen, Emily Oken, Adriëtte J. J. M. Oostvogels, Agnieszka Pac, Eleni Papadopoulou, Juha Pekkanen, Costanza Pizzi, Kinga Polanska, Daniela Porta, Lorenzo Richiardi, Sheryl L. Rifas-Shiman, Nel Roeleveld, Loreto Santa-Marina, Ana C. Santos, Henriette A. Smit, Thorkild I. A. Sørensen, Marie Standl, Maggie Stanislawski, Camilla Stoltenberg, Elisabeth Thiering, Carel Thijs, Maties Torrent, Suzanne C. Tough, Tomas Trnovec, Marleen M. H. J. van Gelder, Lenie van Rossem, Andrea von Berg, Martine Vrijheid, Tanja G. M. Vrijkotte, Oleksandr Zvinchuk, Stef van Buuren, Vincent W. V. Jaddoe

**Affiliations:** 1 The Generation R Study Group, Erasmus MC, University Medical Center Rotterdam, PO Box 2040, 3000 CA Rotterdam, the Netherlands; 2 Department of Pediatrics, Sophia Children’s Hospital, Erasmus MC, University Medical Center Rotterdam, Rotterdam, the Netherlands; 3 TNO Child Health, Leiden, the Netherlands; 4 Department of Epidemiology and Biostatistics, VU University Medical Center, Amsterdam, the Netherlands; 5 EPIUnit-Instituto de Saúde Pública, Universidade do Porto, Rua das Taipas, n° 135, 4050-600 Porto, Portugal; 6 Department of Public Health and Forensic Sciences and Medical Education, Unit of Clinical Epidemiology, Predictive Medicine and Public Health, University of Porto Medical School, Porto, Portugal; 7 INSERM, UMR1153 Epidemiology and Biostatistics Sorbonne Paris Cité Center (CRESS), ORCHAD Team, Villejuif, France; 8 Paris Descartes University, Villejuif, France; 9 Department of Preventive Medicine, Keck School of Medicine, University of Southern California, Los Angeles, CA USA; 10 Department of Social Medicine, Faculty of Medicine, University of Crete, Heraklion, Greece; 11 Department of Genetics and Cell Biology, Maastricht University, Maastricht, the Netherlands; 12 Inserm UMR 1085, Irset-Research Institute for Environmental and Occupational Health, F-35000 Rennes, France; 13 First Department of Pediatrics, Athens University Medical School, Aghia Sophia Children’s Hospital, National and Kapodistrian University of Athens, Athens, Greece; 14 Department of Epidemiology, University Medical Center Groningen, University of Groningen, P.O. Box 30.001, 9700 RG Groningen, the Netherlands; 15 MRC Lifecourse Epidemiology Unit, University of Southampton, Southampton, UK; 16 Centre de Recherche du Centre Hospitalier de l’Universite de Sherbrooke, Sherbrooke, QC Canada; 17 Department of Exposure and Environmental Epidemiology, Norwegian Institute of Public Health, Oslo, Norway; 18 The Department of Biomedical and Neuromotor Sciences, University of Bologna, Bologna, Italy; 19 Department of Epidemiology, Lazio Regional Health Service, Rome, Italy; 20 Department of Woman and Child Health, Ospedale Versilia, Local Health Authority Toscana Nord Ovest, Viareggio, Italy; 21 NIHR Southampton Biomedical Research Centre, University of Southampton and University Hospital Southampton NHS Foundation Trust, Southampton, UK; 22 Division of Metabolic and Nutritional Medicine, Dr. von Hauner Children’s Hospital, Ludwig-Maximilian-Universität Munich, 80337 Munich, Germany; 23 Department of Environmental Epidemiology, Nofer Institute of Occupational Medicine, Lodz, Poland; 24 Department of Public Health Sciences, School of Medicine, University of California Davis, Davis, CA 95616 USA; 25 Department of Population Medicine, Harvard Medical School, Harvard Pilgrim Health Care Institute, Boston, MA USA; 26 Diabetes Unit, Massachusetts General Hospital, Boston, MA USA; 27 Center for Global Health, University of Illinois College of Medicine, Chicago, IL USA; 28 Telethon Kids Institute, The University of Western Australia, Perth, WA Australia; 29 Departments of Public Health Sciences and Environmental Medicine, University of Rochester School of Medicine and Dentistry, Rochester, NY USA; 30 Department of Health Security, National Institute for Health and Welfare, Kuopio, Finland; 31 MRC Integrative Epidemiology Unit, University of Bristol, Oakfield House, Oakfield Grove, Bristol, BS8 2BN UK; 32 Population Health Science, Bristol Medical School, University of Bristol, Bristol, BS8 2BN UK; 33 Department of Public Health, University of Turku, Turku, Finland; 34 Department of Environmental Immunology/Core Facility Studies, Helmholtz Centre for Environmental Research–UFZ, Leipzig, Germany; 35 Epidemiology and Environmental Health Joint Research Unit, FISABIO−Universitat Jaume I−Universitat de València, Valencia, Spain; 36 CIBER Epidemiología y Salud Pública (CIBERESP), Madrid, Spain; 37 Division of Health Data and Digitalization, Norwegian Institute of Public Health, Oslo, Norway; 38 Department of Epidemiology, Chair of Epidemiology and Preventive Medicine, Jagiellonian University Medical College, Krakow, Poland; 39 Turku Centre for Biotechnology, University of Turku and Abo Akademi University, Turku, Finland; 40 Department of Nutrition and Dietetics, School of Health Science and Education, Harokopio University, Athens, Greece; 41 Department of Pediatrics, Cumming School of Medicine, University of Calgary, Calgary, Alberta Canada; 42 Department of Epidemiology, Care and Public Health Research Institute, Maastricht University, Maastricht, the Netherlands; 43 National Institute of Public Health, University of Southern Denmark, Copenhagen, Denmark; 44 Department of Public Health, Section of Epidemiology, University of Copenhagen, Øster Farimagsgade 5, 1014 Copenhagen, Denmark; 45 Department of Rehabilitation, Nutrition and Sport, La Trobe University, Melbourne, Australia; 46 Department of Environmental Medicine, Faculty of Public Health, Slovak Medical University, Bratislava, Slovak Republic; 47 School of Women’s and Infants’ Health, University of Western Australia, Crawley, Western Australia Australia; 48 Research Unit for Gynaecology and Obstetrics, Institute for Clinical Research, University of Southern Denmark, Odense, Denmark; 49 Department of Public Health, Amsterdam Public Health Research Institute, Academic Medical Center, Amsterdam, the Netherlands; 50 Department of Environmental Exposures and Epidemiology, Domain of Infection Control and Environmental Health, Norwegian Institute of Public Health, Lovisenberggata 8, 0477 Oslo, Norway; 51 Department of Public Health, University of Helsinki, Helsinki, Finland; 52 Department of Medical Sciences, University of Turin, Turin, Italy; 53 Department for Health Evidence, Radboud Institute for Health Sciences, Radboud University Medical Center, Nijmegen, the Netherlands; 54 Subdirección de Salud Pública Gipuzkoa, San Sebastián, Spain; 55 Instituto de Investigación Sanitaria BIODONOSTIA, San Sebastián, Spain; 56 Julius Center for Health Sciences and Primary Care, University Medical Center Utrecht, Utrecht University, Utrecht, the Netherlands; 57 The Novo Nordisk Foundation Center for Basic Metabolic Research, Section of Metabolic Genetics, Faculty of Health and Medical Sciences, University of Copenhagen, Copenhagen, Denmark; 58 Institute of Epidemiology, Helmholtz Zentrum München-German Research Center for Environmental Health, Neuherberg, Germany; 59 School of Public Health, University of Colorado, Aurora, Colorado USA; 60 Norwegian Institute of Public Health, Oslo, Norway; 61 Department of Global Public Health and Primary Care, University of Bergen, Bergen, Norway; 62 Dr. von Hauner Children’s Hospital, Ludwig-Maximilians-University Munich, Munich, Germany; 63 Ib-salut, Area de Salut de Menorca, Menorca, Spain; 64 Department of Community Health Sciences, Cumming School of Medicine, University of Calgary, Calgary, Alberta Canada; 65 Department of Environmental Medicine, Slovak Medical University, Bratislava, 833 03 Slovak Republic; 66 Radboud REshape Innovation Center, Radboud University Medical Center, Nijmegen, the Netherlands; 67 Department of Pediatrics, Marien-Hospital Wesel, Research Institute, Wesel, Germany; 68 ISGlobal, Institute for Global Health, Barcelona, Spain; 69 Universitat Pompeu Fabra (UPF), Barcelona, Spain; 70 Department of Medical and Social Problems of Family Health, Institute of Pediatrics, Obstetrics and Gynecology, Kyiv, Ukraine; 71 Department of Methodology and Statistics, University of Utrecht, Utrecht, the Netherlands; 72 Department of Epidemiology, Erasmus MC, University Medical Center Rotterdam, Rotterdam, the Netherlands

**Keywords:** Weight gain, Pregnancy, Charts, References

## Abstract

**Background:**

Gestational weight gain differs according to pre-pregnancy body mass index and is related to the risks of adverse maternal and child health outcomes. Gestational weight gain charts for women in different pre-pregnancy body mass index groups enable identification of women and offspring at risk for adverse health outcomes. We aimed to construct gestational weight gain reference charts for underweight, normal weight, overweight, and grades 1, 2 and 3 obese women and to compare these charts with those obtained in women with uncomplicated term pregnancies.

**Methods:**

We used individual participant data from 218,216 pregnant women participating in 33 cohorts from Europe, North America, and Oceania. Of these women, 9065 (4.2%), 148,697 (68.1%), 42,678 (19.6%), 13,084 (6.0%), 3597 (1.6%), and 1095 (0.5%) were underweight, normal weight, overweight, and grades 1, 2, and 3 obese women, respectively. A total of 138, 517 women from 26 cohorts had pregnancies with no hypertensive or diabetic disorders and with term deliveries of appropriate for gestational age at birth infants. Gestational weight gain charts for underweight, normal weight, overweight, and grade 1, 2, and 3 obese women were derived by the Box-Cox *t* method using the generalized additive model for location, scale, and shape.

**Results:**

We observed that gestational weight gain strongly differed per maternal pre-pregnancy body mass index group. The median (interquartile range) gestational weight gain at 40 weeks was 14.2 kg (11.4–17.4) for underweight women, 14.5 kg (11.5–17.7) for normal weight women, 13.9 kg (10.1–17.9) for overweight women, and 11.2 kg (7.0–15.7), 8.7 kg (4.3–13.4) and 6.3 kg (1.9–11.1) for grades 1, 2, and 3 obese women, respectively. The rate of weight gain was lower in the first half than in the second half of pregnancy. No differences in the patterns of weight gain were observed between cohorts or countries. Similar weight gain patterns were observed in mothers without pregnancy complications.

**Conclusions:**

Gestational weight gain patterns are strongly related to pre-pregnancy body mass index. The derived charts can be used to assess gestational weight gain in etiological research and as a monitoring tool for weight gain during pregnancy in clinical practice.

**Electronic supplementary material:**

The online version of this article (10.1186/s12916-018-1189-1) contains supplementary material, which is available to authorized users.

## Background

Gestational weight gain is an important predictor of adverse maternal and child health outcomes [1]. Insufficient weight gain is associated with increased risks of preterm birth and delivering a low birth weight infant, whereas excessive weight gain is associated with increased risks of gestational hypertension, preterm birth, delivering a high birth weight infant, cesarean delivery, and childhood overweight [2–5].

Appropriate gestational weight gain charts are necessary to monitor the progress of weight gain and to enable risk selection. Gestational weight gain charts have been derived from country-specific studies that varied in sample selection, study design, and methods of data collection and statistical analysis [6]. A study of the INTERGROWTH-21st Project among 3097 normal weight women from Brazil, China, India, Italy, Kenya, Oman, UK, and USA described the patterns in maternal gestational weight gain from 14 weeks onwards in healthy pregnancies with good maternal and perinatal outcomes [7]. Another previous hospital-based study developed gestational weight gain charts for 4246 overweight and obese US women, respectively, delivering uncomplicated term pregnancies [8]. Also, weight gain for gestational age charts for underweight, normal weight, overweight, and grades 1, 2, and 3 obese women were created in a large population-based cohort of 141,767 Swedish women with term, non-anomalous, singleton pregnancies and no pre-existing hypertension or diabetes [9]. Results from these studies showed the strong influence of pre-pregnancy body mass index (BMI) on gestational weight gain. The generalizability of these charts to other populations is not known. International gestational weight gain charts for specific pre-pregnancy BMI groups are important to improve clinical monitoring and risk selection of pregnant women.

We used individual participant data from 218,216 pregnant women from 33 European, North American, and Oceania pregnancy cohort studies to assess the pattern of weight gain and to construct gestational weight gain charts for underweight, normal weight, overweight, and grades 1, 2, and 3 obese women. Additionally, we compared these charts to those obtained in 138,517 pregnant women from 26 cohorts who had uncomplicated term pregnancies.

## Methods

### Inclusion criteria and participating cohorts

This study was embedded in an international collaboration on Maternal Obesity and Childhood Outcomes (MOCO). Pregnancy and birth cohort studies participated if they included mothers with singleton live-born children born from 1989 onwards, had information available on maternal pre/early-pregnancy BMI and at least one offspring measurement (birth weight or childhood BMI) and were approved by their local institutional review boards. We identified 50 cohorts from Europe, North America, and Oceania selected from the existing collaborations on childhood health (EarlyNutrition Project, CHICOS Project, www.birthcohorts.net assessed until July 2014). We invited these cohorts, of which 39 cohorts agreed to participate, providing data of 239,621 singleton births. Detailed information on these cohorts can be found in www.birthcohorts.net. We included cohorts with information on pre-pregnancy BMI and weight measurements throughout pregnancy with information on the corresponding gestational age (33 cohorts). Per cohort, women were included if they had pre-pregnancy BMI to allow classification into the specific pre-pregnancy BMI groups. Therefore, all women had information on weight at 0 weeks, which refers to pre-pregnancy weight. Since the data were modeled cross-sectionally, no further restriction was applied regarding the weight measurements throughout pregnancy. Our final sample comprised 33 cohorts and 218,216 women who contributed with 679,262 gestational weight measurements, of which 218,216 at 0 weeks and 461,046 throughout pregnancy. Of these women, 9065 (4.2%), 148,697 (68.1%), 42,678 (19.6%), 13,084 (6.0%), 3597 (1.6%), and 1095 (0.5%) were underweight, normal weight, overweight, obese grade 1, obese grade 2, and obese grade 3, respectively (flow chart is given in Additional file 1: Figure S1). Twenty-seven of the 33 cohorts defined themselves as regionally or nationally based studies, four as hospital-based (Co.N.ER, EDEN, GASPII, LUKAS), one as internet users-based (NINFEA), and one as studying selected populations (FCOU). To also obtain the charts in uncomplicated pregnancies, we further restricted our sample to women who had pregnancies with no hypertensive or diabetic disorders and with term deliveries of appropriate for gestational age at birth infants. This sample of uncomplicated term pregnancies comprised 26 cohorts and 138, 517 women, of which 5541, 97,263, 26,320, 7160, 1752, and 481 were underweight, normal weight, overweight, and obese grades 1, 2 and 3, respectively. Anonymized datasets were stored on a single central secured data server with access for the main analysts (SS, IE).

### Maternal anthropometrics

Maternal anthropometrics were measured, derived from clinical records or self-reported (cohort-specific information is shown in Additional file 1: Table S1). Maternal pre-pregnancy BMI was calculated from information on height and weight before pregnancy and was categorized as underweight (< 18.5 kg/m^2^), normal weight (18.5–24.9 kg/m^2^), overweight (25.0–29.9 kg/m^2^), obesity grade 1 (30.0–34.9 kg/m^2^), obesity grade 2 (35.0–39.9 kg/m^2^), and obesity grade 3 (≥ 40.0 kg/m^2^) according to the World Health Organization criteria [10]. Data were obtained on early, mid, and late pregnancy weight as the closest measurement to 13 weeks of gestation (range 6–19.9 weeks of gestation), the closest measurement to 26 weeks of gestation (range 20–31.9 weeks of gestation), and the closest measurement to 40 weeks of gestation (range 32–45 weeks of gestation). For the construction of the charts, we created, in a long data format, one single weight variable with the corresponding gestational age. Then, weight gain was calculated as the difference between the weight at certain gestational age and the pre-pregnancy weight. Cohort-specific information on the methods used to estimate gestational age is shown in Additional file 1: Table S1.

### Statistical analysis

We modeled gestational weight gain by gestational age separately for each maternal pre-pregnancy BMI group to develop the pre-pregnancy BMI group-specific gestational weight gain charts. We had available weight measurements at the start of pregnancy and subsequent weights from 8 weeks onwards. For that reason, we modeled from the week 0 onwards. We initially fitted the model in which each woman had a weight gain of 0 kg at the start of pregnancy (0 weeks), but the lack of variation in the outcome caused severe numerical problems. To address this, we imagined a nudge effect equal to the measurement error of body weight. It is known that measurement error of a single dial measurement is about 0.70 kg [11], so the variance of the gain score is equal to 0.70^2^ + 0.70^2^ = 0.98 kg. For each woman, the weight gain at the start of pregnancy was taken as a random draw from the Gaussian distribution with mean of 0 and variance of 0.98 kg. The size of the measurement error was used since it is theoretically based but any variance could have been applied. We started the modeling using a Box-Cox Cole and Green distribution (Box-Cox normal), which turned out to be too strict to fit the data. Therefore, we fitted the models, separately for each maternal pre-pregnancy BMI group, by the Box-Cox *t* (BCT) method using the generalized additive model for location, scale, and shape (GAMLSS) package in R version 3.3.1 [12]. We used GAMLSS instead of quantile regression since in the latter the centiles are estimated individually and thus may cross, leading to an invalid distribution for the outcome. Additionally, there are no distributional assumptions in quantile regression, which may hamper the estimation of the outer centiles with sufficient precision even when there is enough information at the tails [13]. In the BCT method, the default links from the GAMLSS package, namely, an identity link for the mu and nu parts and a log link for the sigma and tau parts of the model, were used. The BCT method summarizes the distribution in four time-dependent smooth curves representing the median (M-curve), the variation (S-curve), the skewness (L-curve), and the kurtosis (T-curve) [14]. The smoothing family and the amount of smoothing were determined by visual inspection of the worm plots, the fitted centiles, and the *Q* statistics [15, 16]. The worm plots describe salient features of the time-conditional *z* score distribution and aid in finding proper smoothing values for the model [15].The M-curve of the models for weight gain was fitted using B-splines smoothing on gestational age with specified internal breakpoints to define the splines and three degrees which is similar to a cubic spline. Cubic splines smoothing on gestational age was also used for the S-curve, L-curve, and T-curve. The models for the different maternal pre-pregnancy BMI groups were fitted with different internal breakpoints and degrees of freedom for the curves. Model specifications for each BMI group are given in Additional file 1: Table S2. Data were modeled cross-sectionally since taking the correlation between repeated observations of the same individual into account seems to have negligible effects on the location and precision of the centiles [13]. We tested for pre-pregnancy weight as well as cohort and country differences in the models. To confirm that using a more advanced model was justified, we tested for each maternal pre-pregnancy BMI group whether our model had a better fit as compared to a simple linear model using the Bayesian information criterion. We also compared our charts to those obtained, using the same analytical strategy and models, in a sample restricted to women who had uncomplicated term pregnancies.

## Results

### Subject characteristics

Characteristics of the participating pregnancy cohorts are given in Table 1. Overall, the median maternal pre-pregnancy BMI and total gestational weight gain were 22.7 kg/m^2^ (interquartile range 20.8–25.4 kg/m^2^) and 14.0 kg (interquartile range 11.0–17.9 kg), respectively. The number of weight measurements during pregnancy available per participating cohort and per maternal pre-pregnancy BMI group is given in Additional file 1: Table S3. The overall sample size according to gestational age for each maternal pre-pregnancy BMI group is shown in Additional file 1: Figure S2. For the construction of the charts, most weight measurements were available around 15, 30, and 40 weeks of gestation and for normal weight and overweight women.

**Table 1:** Characteristics of the participating pregnancy cohorts (*n* = 218,216)^a^

Cohort name, number of participants, birth years (country)	Maternal pre-pregnancy body mass index (kg/m^2^)	Maternal total gestational weight gain (kg)	Gestational age at birth (weeks)
ABCD, *n* = 7820, 2003–2004 (The Netherlands)	22.3 (20.5, 24.8)	NA	40.0 (39.0, 41.0)
ALSPAC, *n* = 11,344, 1991–1992 (UK)	22.2 (20.5, 24.4)	12.5 (9.5, 15.5)	40.0 (39.0, 41.0)
AOB/F, *n* = 2941, 2008–2010 (Canada)	23.0 (20.8, 26.3)	NA	39.0 (38.0, 40.0)
Co.N.ER, *n* = 637, 2004–2005 (Italy)	21.1 (19.7, 23.4)	13.0 (10.0, 16.0)	39.0 (39.0, 40.0)
DNBC, *n* = 42,761, 1996–2002 (Denmark)^b^	22.5 (20.7, 25.1)	15.0 (12.0, 18.0)	40.1 (39.1, 41.0)
EDEN, *n* = 1875, 2003–2005 (France)	22.1 (20.1, 25.3)	13.0 (11.0, 16.3)	39.0 (39.0, 40.0)
FCOU, *n* = 3650, 1993–1996 (Ukraine)	21.6 (19.8, 24.0)	12.0 (9.2, 15.0)	40.0 (39.0, 41.0)
GASPII, *n* = 675, 2003–2004 (Italy)	21.3 (19.8, 23.6)	13.0 (10.5, 16.0)	40.0 (39.0, 41.0)
GECKO Drenthe, *n* = 2501, 2006–2007 (The Netherlands)	23.7 (21.5, 26.8)	13.0 (10.0, 17.0)	40.0 (39.0, 40.9)
Generation R, *n* = 7183 2002–2006 (The Netherlands)	22.6 (20.8, 25.4)	12.0 (9.0, 16.0)	40.1 (39.0, 41.0)
Generation XXI, *n* = 7621, 2005–2006 (Portugal)	22.9 (21.0, 25.8)	13.0 (10.0, 17.0)	39.0 (38.0, 40.0)
GENESIS, *n* = 2218, 2003–2004 (Greece)	21.9 (20.2, 24.0)	13.0 (10.0, 17.0)	40.0 (39.0, 40.0)
Gen3G, *n* = 846, 2010–2013 (Canada)	23.3 (20.9, 27.3)	13.7 (10.7, 17.0)	39.4 (38.5, 40.2)
GINIplus, *n* = 2329, 1995–1998 (Germany)	22.1 (20.4, 24.2)	13.0 (10.0, 15.7)	40.0 (39.0, 41.0)
HUMIS, *n* = 1067, 2003–2008 (Norway)	23.5 (21.3, 26.2)	14.0 (11.0, 18.0)	40.1 (39.0, 41.1)
INMA, *n* = 2561, 1997–2008 (Spain)	22.5 (20.7, 25.0)	13.5 (10.5, 16.6)	39.9 (38.9, 40.6)
KOALA, *n* = 2812, 2000–2002 (The Netherlands)	22.7 (20.9, 25.3)	14.0 (11.0, 17.0)	40.0 (39.0, 40.0)
Krakow Cohort, *n* = 503, 2000–2003 (Poland)	21.0 (19.5, 22.7)	15.0 (12.0, 18.0)	40.0 (39.0, 40.0)
LISAplus, *n* = 2962, 1997–1999 (Germany)	21.7 (20.2, 24.1)	14.0 (11.5, 17.0)	40.0 (39.0, 41.0)
LUKAS, *n* = 417, 2002–2005 (Finland)	24.1 (21.9, 27.2)	13.8 (10.9, 17.8)	40.0 (39.0, 40.0)
MoBa, *n* = 88,503, 1999–2009 (Norway)	23.1 (21.1, 25.9)	15.0 (11.0, 18.0)	40.1 (39.1, 41.0)
NINFEA, *n* = 2237, 2005–2010 (Italy)^c^	21.4 (19.9, 23.9)	12.0 (10.0, 15.0)	39.7 (38.9, 40.7)
PÉLAGIE, *n* = 1490, 2002–2005 (France)	21.6 (20.0, 23.8)	NA	40.0 (39.0, 40.0)
PIAMA, *n* = 3459, 1996–1997 (The Netherlands)	22.2 (20.6, 24.3)	13.0 (10.0, 16.0)	40.0 (39.1, 40.9)
Piccolipiù, *n* = 3294, 2011–2015 (Italy)	21.7 (19.9, 24.2)	13.0 (10.0, 15.0)	39.0 (39.0, 40.0)
PRIDE Study, *n* = 1513, 2011–2015 (The Netherlands)	22.5 (20.7, 24.8)	14.0 (11.0, 17.0)	39.0 (39.0, 40.0)
Project Viva, *n* = 2106, 1999–2002 (United States)	23.5 (21.0, 27.3)	15.5 (12.3, 19.1)	39.7 (38.9, 40.6)
Raine Study, *n* = 2791, 1989–1992 (Australia)	21.3 (19.6, 23.7)	NA	39.0 (38.0, 40.0)
REPRO_PL, *n* = 1409, 2007–2011(Poland)	21.5 (19.8, 23.8)	12.0 (9.0, 15.0)	39.0 (38.5, 40.0)
RHEA, *n* = 816, 2007–2008 (Greece)	23.3 (21.2, 26.2)	13.0 (10.0, 17.0)	38.0 (38.0, 39.0)
Slovak PCB study, *n* = 1048, 2002–2004 (Slovakia)	21.2 (19.5, 24.0)	13.0 (10.0, 17.0)	40.0 (39.0, 40.0)
STEPS, *n* = 1708, 2008–2010 (Finland)	23.0 (21.1, 26.1)	13.9 (10.8, 17.4)	40.0 (39.0, 41.0)
SWS, *n* = 3119, 1998–2007 (UK)	24.1 (21.9, 27.4)	11.9 (8.3, 15.7)	40.0 (39.0, 41.0)
Total group	22.7 (20.8, 25.4)	14.0 (11.0, 17.9)	40.0 (39.0, 41.0)

^a^Values are expressed as medians (interquartile range). *NA* not available

^b^Subset of participants with offspring body mass index available at 7 years by the time of data transfer (May 2015)

^c^Subset of participants with follow-up completed at 4 years of child’s age by the time of data transfer (March 2015)

### Gestational weight gain charts

**Fig. 1:**
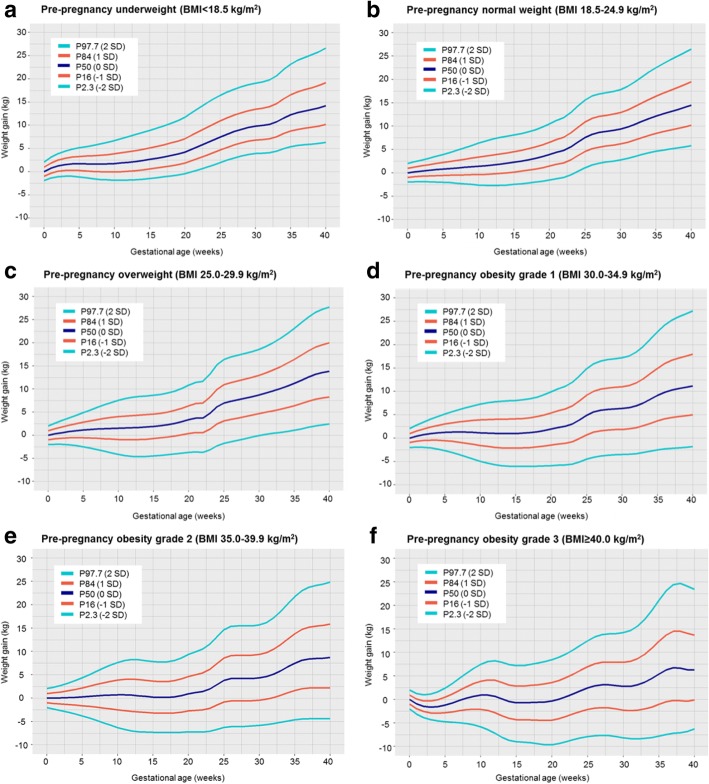
Selected percentiles of weight gain for gestational age for maternal pre-pregnancy underweight (**a**), normal weight (**b**), overweight (**c**), obesity grade 1 (**d**), obesity grade 2 (**e**) and obesity grade 3 (**f**)

**Fig. 2:**
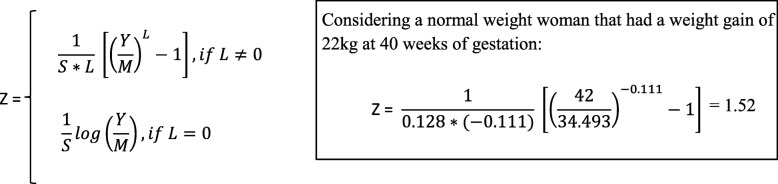
Equation for the calculation of pre-pregnancy body mass index-specific gestational weight gain *z* scores based on a Box-Cox *t* model^a^. ^**a**^where *Y* is weight gain at a certain gestational age, *L* is lambda, *M* is mu, and *S* is sigma. The random variable *Z* is assumed to follow a *t* distribution with degrees of freedom, Tau > 0, treated as a continuous parameter. The parameters of our Box-Cox *t* model for each pre-pregnancy body mass index group are provided for the rounded gestational ages. This equation can be applied on data using the y2z function of the AGD package in R. The function will allow the calculation of *z* scores for the exact gestational age by extrapolating the parameters. For applying the equation or function, weight gain must be > 0, because the model cannot deal with negative values. In order to fit the Box-Cox *t* model, parameters were calculated based on weight gain + 20 kg, and thus 20 kg must be added to weight gain to be able to use our parameters. The constant of 20 kg was chosen since − 20 kg is an extremely low value for weight change during pregnancy. After adding the 20 kg, weight gain must be > 0; otherwise, the equation or function using our Box-Cox *t* model parameters cannot be applied for the remaining ≤ 0 values

Figure 1 shows selected percentiles of weight gain for gestational age (P2.3 (− 2 SD), P16 (− 1 SD), P50 (0 SD), P84 (1 SD), and P97.7 (2 SD)) for underweight, normal weight, overweight, and grades 1, 2, and 3 obese women. Gestational weight gain strongly differed per maternal pre-pregnancy BMI group and was gradually lower across higher BMI groups. The median (interquartile range) gestational weight gain at 40 weeks was 14.2 kg (11.4–17.4) for underweight women; 14.5 kg (11.5–17.7) for normal weight women; 13.9 kg (10.1–17.9) for overweight women; and 11.2 kg (7.0–15.7), 8.7 kg (4.3–13.4), and 6.3 kg (1.9–11.1) for grades 1, 2, and 3 obese women, respectively. For all maternal pre-pregnancy BMI groups, weight gain trajectories throughout pregnancy followed a non-linear shape. The Bayesian information criterion supported our non-linear model that showed a better statistical fit as compared to a simple linear model. The rate of weight gain was lower in the first half than in the second half of pregnancy for all pre-pregnancy BMI groups. Especially in overweight women, we observed a higher rate of weight gain around 22–25 weeks of gestation. The coefficients of variation between pre-pregnancy weights within the same BMI group, and between cohorts and countries were smaller than the measurement error (variance of the weight gain of 0.98 kg), reinforcing the similarities in the charts for the variety of weights within each BMI group and among cohorts and countries. These findings also suggest no strong cohort birth period or region effects on our charts. The predicted *z* scores for the average weight gain according to gestational age for each maternal BMI group are shown in Additional file 1: Figure S3. Only a small misfit, caused by less data available, was observed for grade 3 obese women. Estimates of weight gain for selected percentiles according to gestational age and maternal BMI groups are given in Additional file 1: Tables S4-S9. Figure 2 shows the equation for the calculation of *z* scores based on a BCT model. The parameters of our BCT model at a certain gestational age to allow the calculation of *z* scores are given in Additional file 1: Tables S4-S9 (available in an excel spreadsheet upon request). An online tool to produce individual *z* scores and percentiles for gestational weight gain in singleton pregnancies based on our international reference charts is available at https://lifecycle-project.eu.

**Fig. 3:**
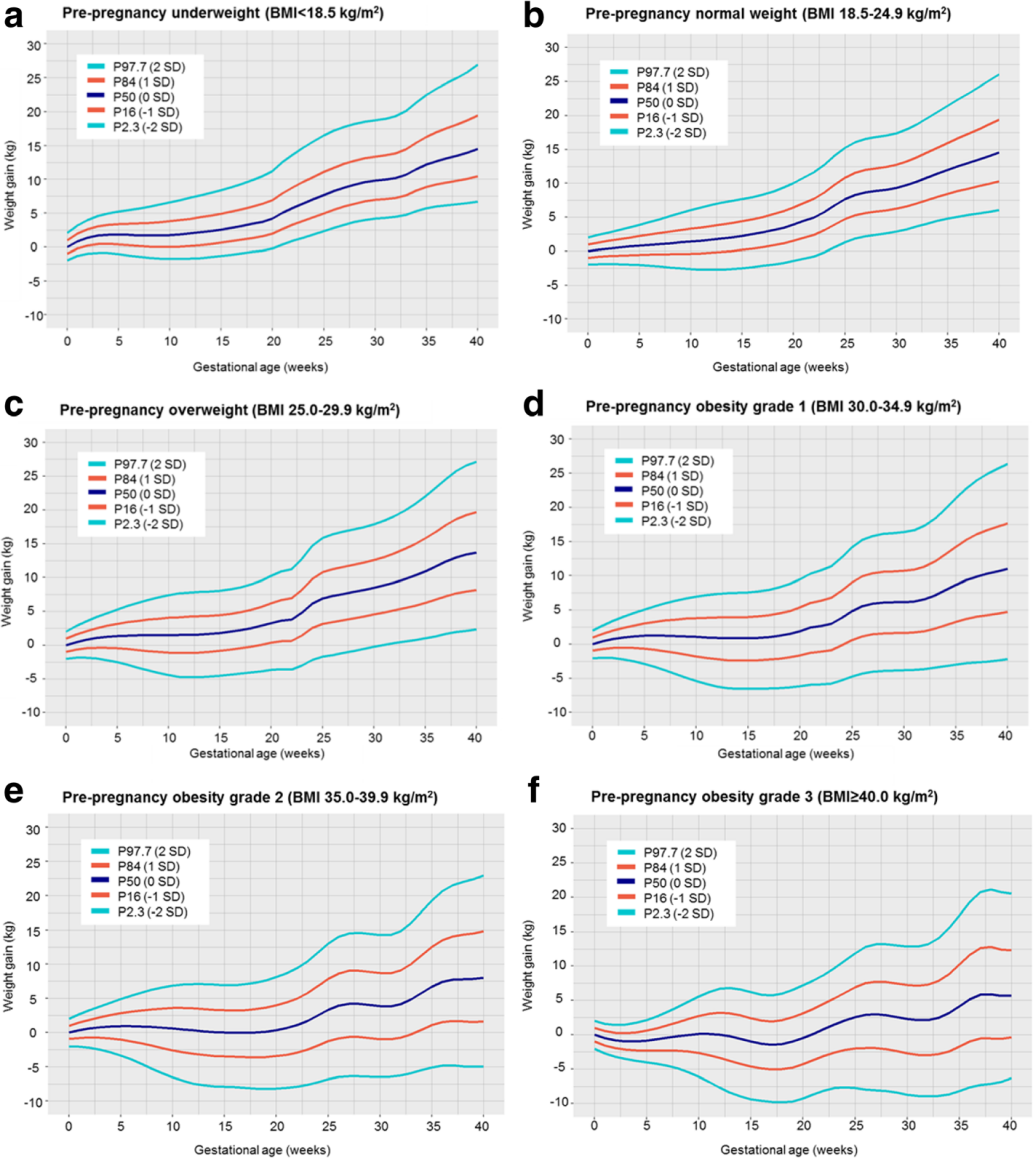
Selected percentiles of weight gain for gestational age in women without any pregnancy complication for maternal pre-pregnancy underweight (**a**), normal weight (**b**), overweight (**c**), obesity grade 1 (**d**), obesity grade 2 (**e**) and obesity grade 3 (**f**)

Similar charts were obtained when we applied the same models to a sample without pregnancy complications (Fig. 3). We also observed similar estimates of weight gain for P50 at 20 and 40 weeks of gestation for all maternal pre-pregnancy BMI groups in all pregnant women and in women without any pregnancy complication. Although the estimates were largely similar, we observed that women without any pregnancy complication who were underweight or normal weight tended to gain higher weight and those who were overweight or obese tended to gain lower weight, compared to the full group of pregnant women (Table 2). Similar results were observed when restricting all analyses to the regionally and nationally based cohorts (data not shown).

**Table 2:** Percentile 50 of gestational weight gain at 20 and 40 weeks for maternal pre-pregnancy body mass index groups in all pregnant women and in women without any pregnancy complication

	P50 of weight gain (kg) at 20 weeks		P50 of weight gain (kg) at 40 weeks	
	All pregnant women	Women without any pregnancy complication	All pregnant women	Women without any pregnancy complication
Underweight	4.20	4.17	14.20	14.47
Normal weight	3.90	3.91	14.49	14.53
Overweight	3.35	3.28	13.86	13.68
Obesity grade 1	1.95	1.93	11.19	10.99
Obesity grade 2	0.93	0.34	8.73	8.02
Obesity grade 3	−0.35	−0.49	6.27	5.65

## Discussion

In this study, we developed gestational weight gain charts for different pre-pregnancy BMI groups for women in Europe, North America, and Oceania. Gestational weight gain strongly differed per maternal pre-pregnancy BMI group and was gradually lower across higher BMI groups. For all maternal BMI groups, weight gain throughout pregnancy followed a non-linear trajectory. The rate of weight gain was greater in the second than in the first half of pregnancy. No differences in the patterns of weight gain were observed between cohorts or countries. Our reference charts were largely similar to those obtained in a sample restricted to uncomplicated term pregnancies.

### Interpretation of main findings

Gestational weight gain is an important predictor of adverse maternal and child health outcomes [1]. Weight gain reflects multiple components. It has been suggested that about 30% of gestational weight gain comprises the fetus, amniotic fluid, and placenta, whereas the remaining 70% comprises uterine and mammary tissue expansion, increased blood volume, extracellular fluid, and fat stores [17]. The US Institute of Medicine (IOM) published in 2009 the revised recommended gestational weight gain ranges, i.e., 12.5–18 kg, 11.5–16 kg, 7–11.5 kg, and 5–9 kg for underweight, normal weight, overweight, and obese women, respectively, based on findings from observational studies focused on associations of gestational weight gain with preterm birth, small, and large size for gestational age at birth, cesarean delivery, postpartum weight retention, and childhood obesity [1]. Both insufficient and excessive gestational weight gain, defined according to these guidelines, are risk factors of adverse maternal and child health outcomes [2–5]. In our study, insufficient, adequate, and excessive gestational weight gain was observed in 38.1%, 43.8%, and 18.1% of underweight women; 25.4%, 41.5%, and 33.1% of normal weight women; 9.8%, 24.3%, and 65.9% of overweight women; and 18.6%, 24.0%, and 57.4% of obese women, respectively.

Gestational weight gain charts are important from a clinical and epidemiological perspective. From a clinical perspective, appropriate gestational weight gain charts can help to identify individuals at risk for adverse health outcomes. It has been recognized that it might be problematic to link total gestational weight gain with pregnancy outcomes that are highly correlated with gestational age at birth, such as preterm birth. Women who deliver at earlier gestational ages have less time to gain weight, which may lead to a spurious association between low gestational weight gain and preterm birth. The use of the rate of weight gain (kg per week of gestation) reduces but does not entirely resolve this bias [2]. Weight gain for gestational age *z* score charts can be used to classify weight gain independently of gestational age and provide a tool to establish the unbiased associations between gestational weight gain and pregnancy outcomes. This method enables comparison of weight gain of women who deliver at earlier gestational ages with weight gain of women with normal pregnancy duration at the same point in pregnancy. Although various gestational weight gain charts have previously been developed, these charts vary across different studies and still have methodological limitations [7–9, 18–29]. Based on a recent systematic review of 12 studies involving 2,268,556 women from 9 countries, differences in the methodological quality of gestational weight gain studies may explain the varying chart recommendations. These charts were all derived from country-specific studies that varied in sample selection, study design, methods of data collection, and statistical analysis [6]. A study among 3097 normal weight women from Brazil, China, India, Italy, Kenya, Oman, UK, and USA described the patterns in maternal gestational weight gain from 14 weeks onwards in healthy pregnancies with good maternal and perinatal outcomes. The authors suggested that weight gain follows a linear trajectory throughout pregnancy, which was similar across the eight populations [7]. A hospital-based study developed gestational weight gain charts for 1047, 1202, 1267, and 730 overweight, grades 1, 2, and 3 obese US women, respectively, delivering uncomplicated term pregnancies. The rate of weight gain was minimal until 15–20 weeks and then increased in a slow, linear manner until term. The rate of weight gain was lower as BMI increased [8]. In a study among 141,767 Swedish women with term, non-anomalous, singleton pregnancies and no pre-existing hypertension or diabetes, the rate of weight gain also decreased with increasing BMI. In normal weight, overweight and grade 1 obese women, the median rate of weight gain was minimal until 15 weeks, after which it increased in a linear manner until term whereas in underweight, and grades 2 and 3 obese women, the median rate of weight gain was steady throughout gestation [9]. The generalizability of these charts to other populations is not known.

In the current study, we constructed gestational weight gain reference charts for 218,216 underweight, normal weight, overweight, and grades 1, 2, and 3 obese women using data from cohorts from Europe, North America, and Oceania. We observed that for all maternal pre-pregnancy BMI groups, weight gain throughout pregnancy followed a non-linear trajectory. This finding is not consistent with results of previous studies that suggested that weight gain follows a linear trajectory at least from the second half of pregnancy onwards [7–9]. We included a large spectrum of gestational age and had a large number of participants and weight measurements available, enabling the detection of small variations in the weight gain patterns. The non-linearity of the trajectories was supported by advanced visual diagnostic methods for model choice and information criteria. This difference in the pattern of weight gain between our study and previous studies is not a result of longitudinal or cross-sectional modeling since the inclusion of the correlation structure among observations seems to have negligible effects on the location and precision of the centiles [13]. Therefore, from a statistical point of view, we believe that these charts describe the actual track of weight gain during pregnancy and that a simpler method assuming a linear weight gain fits the data less well. From a biological point of view, gestational weight gain reflects multiple fetal and maternal components [17]. This non-linearity might be the result of fluctuations in these components throughout pregnancy. This variation in the weight gain seems to be more pronounced in the obese groups. Also, contributing to this non-linearity, we observed a greater rate of weight gain around 22–25 weeks, especially in overweight women, which might be related to the initiation of adipose tissue formation in the fetus that is known to occur between the 14th and the 23rd week of gestation [30]. In the current study, the rate of weight gain was greater in the second than in the first half of pregnancy and was lower as pre-pregnancy BMI was higher. Despite the range of cultures, behaviors, clinical practices, and traditions, which can strongly influence gestational weight gain, we did not observe differences in the patterns of weight gain between cohorts and countries. This finding might indicate that the biological process of gaining weight during pregnancy does not differ across different international populations in Europe, North America, and Oceania.

Gestational weight gain charts can be classified as reference charts or standard charts. A reference chart is based on a sample of the general population and is descriptive, whereas a standard chart is only focused on a healthy population and is prescriptive. The use of references or standards might influence the chart recommendations. Gestational weight gain standards might be biased by the definition of what constitutes a healthy population, especially for overweight and obese women, and might be compromised by an inadequate sample size. The INTERGROWTH-21st Project developed standards in an international population of normal weight women by only including women with healthy pregnancies with good maternal and perinatal outcomes [7]. However, a recent study showed that the INTERGROWTH-21st standards do not seem to describe optimal weight gain patterns with respect to maternal postpartum weight retention and thus may still be descriptive [31]. We developed gestational weight gain reference charts by including all pregnant women that had all necessary information available for these analyses and compared with the charts obtained in a sample with good maternal and perinatal outcomes. We observed similar weight gain patterns for each maternal BMI group in all pregnant women and in women without any pregnancy complication. Thus, our reference charts are largely similar to those obtained in a sample restricted to uncomplicated term pregnancies, were developed in a large sample, enabling relatively accurate charts for women with severe obesity, and were less likely to bias in the definition of the population. We consider our reference charts as appropriate charts for clinical practice and epidemiological research. However, future studies are needed to relate the derived reference charts to maternal and offspring outcomes and to create customized weight gain charts by including factors such as parity and ethnicity. Finally, since the causality for the associations of maternal gestational weight gain with maternal and child’s health outcomes remain unclear, practicing prenatal care on weight gain is still debatable [32, 33]. A further unanswered question is whether alteration of these gestational weight gain patterns is achievable as, to date, randomized controlled trials focused on lifestyle interventions during pregnancy have shown only small reductions in gestational weight gain [33–35].

### Strengths and limitations

Strengths of this study were the description of the pattern of weight gain throughout pregnancy in a large sample of pregnant women from 33 cohorts from Europe, North America, and Oceania. However, our chart for grade 3 obese women would have benefited from a larger sample and thus the values of selected percentiles in our chart may differ from the true values in the underlying population. We included data from cohort studies from Europe, North America, and Oceania but a large proportion of data come from Northern Europe. This suggests that our charts might be generalizable to Western populations and specifically to populations of Northern European ancestry. Further studies are needed to develop gestational weight gain charts among populations from low- to middle-income countries and of different ethnic backgrounds. Since most studies were general population-based cohort studies, we might have an overrepresentation of the healthier population due to selective non-response in the participating cohorts. This might have underestimated the prevalence of inadequate and excessive gestational weight gain and of the adverse health outcomes. However, we observed similar findings in the full group and when we restricted our analyses to women with uncomplicated pregnancies, which suggest no strong bias due to selection in the cohorts. Also, due to the data request format within this collaboration, only one weight measurement at early, mid, and late pregnancy was obtained, when available, for each woman even if multiple weight measurements were taken during each period. This might have limited the number of weight measurements available for the creation of these charts. For our analyses, we had available weight measurements at the start of pregnancy and subsequent weights from 8 weeks onwards. The lack of weight measurements during the beginning of pregnancy could have influenced the modeling of weight gain patterns, but we believe this is unlikely since not much variation is expected during this period. The correlation between weight at the start of pregnancy and weight at 8 weeks of gestation was 0.99 and an intraclass correlation coefficient using an absolute agreement definition of 97.9% was obtained through a two-way mixed effects model. Finally, we relied not only on weight data obtained by measurements and derived from clinical records but also on self-reported data, which might be a source of error. Women tend to underestimate their weight on self-report [36]. An underestimation of pre-pregnancy weight might lead to a misclassification of women in the different BMI groups and to an overestimation of weight gain at each specific week of gestation. Since measured pre-pregnancy weight is rarely available in routine clinical practice, our reference charts reflect the information usually used to assess weight gain in the prenatal care. Methods of gestational age assessment might also be prone to error, leading to some inaccuracy in the gestational weight gain percentiles and *z* scores, though the error in gestational age estimates and thus the influence on our results is likely to be small. For the construction of the standards, we excluded women based on direct pregnancy-related complications, such as hypertensive or diabetic disorders, preterm deliveries, and small or large for gestational age at birth infants. Unfortunately, information about excess postpartum weight retention and infant deaths was not available.

## Conclusions

We developed gestational weight gain reference charts for different pre-pregnancy BMI groups for women in Europe, North America, and Oceania. Gestational weight gain strongly differed per maternal pre-pregnancy BMI group and was gradually lower across higher BMI groups. These reference charts can be used to classify weight gain independently of gestational age in etiological research focused on maternal and offspring consequences of weight gain. Future research is needed that relates these charts with a broad range of maternal and child health outcomes. These charts may be useful in clinical practice to identify women at risk for adverse short- and long-term health outcomes.

## Additional file

Additional file 1: **Figure S1.** Flow chart of participating cohorts and individuals. **Table S1.** Cohort-specific methods of data collection for maternal anthropometrics and gestational age. **Table S2.** Box-Cox *t* model specifications for each maternal pre-pregnancy body mass index group. **Table S3.** Gestational weight measurements per participating cohort and maternal pre-pregnancy body mass index group.** Figure S2.** Sample size according to gestational age for each maternal pre-pregnancy body mass index group. **Figure S3.** Predicted *z* scores for the average weight gain according to gestational age for each maternal pre-pregnancy body mass index group. **Table S4.** Week-specific Box-Cox *t* model parameters and selected percentiles of gestational weight gain for maternal pre-pregnancy underweight. **Table S5. **Week-specific Box-Cox *t* model parameters and selected percentiles of gestational weight gain for maternal pre-pregnancy normal weight. **Table S6.** Week-specific Box-Cox *t* model parameters and selected percentiles of gestational weight gain for maternal pre-pregnancy overweight. **Table S7.** Week-specific Box-Cox *t* model parameters and selected percentiles of gestational weight gain for maternal pre-pregnancy obesity grade 1. **Table S8.** Week-specific Box-Cox *t* model parameters and selected percentiles of gestational weight gain for maternal pre-pregnancy obesity grade 2. **Table S9.** Week-specific Box-Cox *t* model parameters and selected percentiles of gestational weight gain for maternal pre-pregnancy obesity grade 3. **Table S10.** Local institutional ethical review boards per cohort. (DOCX 631 kb)
