# Supplementary material for: Gestational weight gain charts for different body mass index groups for women in Europe, North America, and Oceania
Source: BMC Med. 2018 Nov 5;16:201. doi: 10.1186/s12916-018-1189-1 (PMC6217770; doi:10.1186/s12916-018-1189-1)
Supplement: Supplementary file 1 — Figure S1. Flow chart of participating cohorts and individuals. Table S1. Cohort-specific methods of data collection for maternal anthropometrics and gestational age. Table S2. Box-Cox t model specifications for each maternal pre-pregnancy body mass index group. Table S3. Gestational weight measurements per participating cohort and maternal pre-pregnancy body mass index group. Figure S2. Sample size according to gestational age for each maternal pre-pregnancy body mass index group. Figure S3. Predicted z scores for the average weight gain according to gestational age for each maternal pre-pregnancy body mass index group. Table S4. Week-specific Box-Cox t model parameters and selected percentiles of gestational weight gain for maternal pre-pregnancy underweight. Table S5. Week-specific Box-Cox t model parameters and selected percentiles of gestational weight gain for maternal pre-pregnancy normal weight. Table S6. Week-specific Box-Cox t model parameters and selected percentiles of gestational weight gain for maternal pre-pregnancy overweight. Table S7. Week-specific Box-Cox t model parameters and selected percentiles of gestational weight gain for maternal pre-pregnancy obesity grade 1. Table S8. Week-specific Box-Cox t model parameters and selected percentiles of gestational weight gain for maternal pre-pregnancy obesity grade 2. Table S9. Week-specific Box-Cox t model parameters and selected percentiles of gestational weight gain for maternal pre-pregnancy obesity grade 3. Table S10. Local institutional ethical review boards per cohort. (DOCX 631 kb) [file 12916_2018_1189_MOESM1_ESM.docx]

**Gestational weight gain charts for different body mass index groups for women in Europe, North America and Oceania**

Susana Santos, Iris Eekhout, Ellis Voerman, Romy Gaillard, Henrique Barros, Marie-Aline Charles, Leda Chatzi, Cécile Chevrier, George P. Chrousos, Eva Corpeleijn, Nathalie Costet, Sarah Crozier, Myriam Doyon, Merete Eggesbø, Maria Pia Fantini, Sara Farchi, Francesco Forastiere, Luigi Gagliardi, Vagelis Georgiu, Keith M. Godfrey, Davide Gori, Veit Grote, Wojciech Hanke, Irva Hertz-Picciotto, Barbara Heude, Marie-France Hivert, Daniel Hryhorczuk, Rae-Chi Huang, Hazel Inskip, Todd A. Jusko, Anne M. Karvonen, Berthold Koletzko, Leanne K. Küpers, Hanna Lagström, Debbie A. Lawlor, Irina Lehmann, Maria-Jose Lopez-Espinosa, Per Magnus, Renata Majewska, Johanna Mäkelä, Yannis Manios, Sheila W. McDonald, Monique Mommers, Camilla S. Morgen, George Moschonis, Ľubica Murínová, John Newnham, Ellen A. Nohr, Anne-Marie Nybo Andersen, Emily Oken, Adriëtte J. J. M. Oostvogels, Agnieszka Pac, Eleni Papadopoulou, Juha Pekkanen, Costanza Pizzi, Kinga Polanska, Daniela Porta, Lorenzo Richiardi, Sheryl L. Rifas-Shiman, Nel Roeleveld, Loreto Santa-Marina, Ana C. Santos, Henriette A. Smit, Thorkild I. A. Sørensen, Marie Standl, Maggie Stanislawski, Camilla Stoltenberg, Elisabeth Thiering, Carel Thijs, Maties Torrent, Suzanne C. Tough, Tomas Trnovec, Marleen M. H. J. van Gelder, Lenie van Rossem, Andrea von Berg, Martine Vrijheid, Tanja G. M. Vrijkotte, Oleksandr Zvinchuk, Stef van Buuren, Vincent W.V. Jaddoe

**Contents**

**Additional Fig 1.** Flow chart of participating cohorts and individuals.

**Additional Table 1.** Cohort-specific methods of data collection for maternal anthropometrics and gestational age.

**Additional Table 2**. Box-Cox *t* model specifications for each maternal pre-pregnancy body mass index group.

**Additional Table 3.** Gestational weight measurements per participating cohort and maternal pre-pregnancy body mass index group.

**Additional Fig 2.** Sample size according to gestational age for each maternal pre-pregnancy body mass index group.

**Additional Fig 3.** Predicted z-scores for the average weight gain according to gestational age for each maternal pre-pregnancy body mass index group.

**Additional Table 4.** Week-specific Box-Cox *t* model parameters and selected percentiles of gestational weight gain for maternal pre-pregnancy underweight.

**Additional Table 5.** Week-specific Box-Cox *t* model parameters and selected percentiles of gestational weight gain for maternal pre-pregnancy normal weight.

**Additional Table 6.** Week-specific Box-Cox *t* model parameters and selected percentiles of gestational weight gain for maternal pre-pregnancy overweight.

**Additional Table 7.** Week-specific Box-Cox *t* model parameters and selected percentiles of gestational weight gain for maternal pre-pregnancy obesity grade 1.

**Additional Table 8.** Week-specific Box-Cox *t* model parameters and selected percentiles of gestational weight gain for maternal pre-pregnancy obesity grade 2.

**Additional Table 9.** Week-specific Box-Cox *t* model parameters and selected percentiles of gestational weight gain for maternal pre-pregnancy obesity grade 3.

**Additional Table 10.** Local institutional ethical review boards per cohort.

**Figure S1.** Flow chart of participating cohorts and individuals

**n = 50** cohorts

Invited

**n = 8** cohorts

No response (n = 4)

Not able to share data (n = 4)

**n = 42** cohorts

Agreed to participate

**n = 3** cohorts

No signed data transfer agreement returned (n = 2)

No dataset received (n = 1)

**n = 39** cohorts

n = 39 signed agreements

n = 39 datasets received

**n = 239,621** individuals

**n = 6** cohorts

**n = 10,954** individuals

No information on maternal pre-pregnancy body mass index or on any weight measurement during pregnancy and corresponding gestational age

**n = 33** cohorts

**n = 228,667** individuals

**n = 10,451** individuals

No information on maternal pre-pregnancy body mass index

**n = 218,216** individuals suitable for analysis

**n = 9,065** underweight

**n = 148,697** normal weight

**n = 42,678** overweight

**n = 13,084** obese grade 1

**n = 3,597** obese grade 2

**n = 1,095** obese grade 3

**Table S1.** Cohort-specific methods of data collection for maternal anthropometrics and gestational age

| **Cohort name (country)** | **Maternal height** | **Maternal pre-pregnancy weight** | **Maternal weights during pregnancy** | **Gestational age at weight measurements** |
| --- | --- | --- | --- | --- |
| ABCD (The Netherlands) | Self-reported | Self-reported | Self-reported | Self-reported |
| ALSPAC (United Kingdom) | Self-reported | Self-reported | Clinical records | Clinical records |
| AOB/F (Canada) | Self-reported | Self-reported | Self-reported | Self-reported |
| Co.N.ER (Italy) | Self-reported | Self-reported | Self-reported | Self-reported |
| DNBC (Denmark) | Self-reported | Self-reported | Self-reported | Self-reported |
| EDEN (France) | Measured | Self-reported | Clinical records | Clinical records |
| FCOU (Ukraine) | Clinical records | Clinical records | Clinical records | Clinical records |
| GASPII (Italy) | Self-reported | Self-reported | Self-reported | Self-reported |
| GECKO Drenthe (The Netherlands) | Self-reported | Self-reported | Self-reported | Clinical records |
| Generation R (The Netherlands) | Measured | Self-reported | Measured | Ultrasound |
| Generation XXI (Portugal) | Measured or copied from the national identity card | Self-reported | Self-reported | Clinical records |
| GENESIS (Greece) | Self-reported | Self-reported | Self-reported | Self-reported |
| Gen3G (Canada) | Measured | Self-reported | Measured | Self-reported or last menstrual period corrected with ultrasound if applicable |
| GINIplus (Germany) | Self-reported | Self-reported | Self-reported | Self-reported |
| HUMIS (Norway) | Self-reported | Self-reported | Self-reported or clinical records | Last menstrual period and ultrasound |
| INMA (Spain) | Measured or self-reported | Self-reported | Measured or clinical records | Last menstrual period and ultrasound |
| KOALA (The Netherlands) | Self-reported | Self-reported | Self-reported | Clinical records |

**Table S1.** Cohort-specific methods of data collection for maternal anthropometrics and gestational age (continued)

| **Cohort name (country)** | **Maternal height** | **Maternal pre-pregnancy weight** | **Maternal weights during pregnancy** | **Gestational age at weight measurements** |
| --- | --- | --- | --- | --- |
| Krakow Cohort (Poland) | Self-reported | Self-reported | Self-reported | Clinical records |
| LISAplus (Germany) | Self-reported | Self-reported | Self-reported | Self-reported |
| LUKAS (Finland) | Self-reported | Self-reported | Clinical records | Clinical records |
| MoBa (Norway) | Self-reported | Self-reported | Self-reported | Self-reported |
| NINFEA (Italy) | Self-reported | Self-reported | Self-reported | Self-reported |
| PÉLAGIE (France) | Self-reported | Self-reported | Self-reported or clinical records | Clinical records or last menstrual period |
| PIAMA (The Netherlands) | Self-reported | Self-reported | Self-reported | Self-reported |
| Piccolipiù (Italy) | Self-reported | Self-reported | Self-reported | Clinical records |
| PRIDE Study (The Netherlands) | Self-reported | Self-reported | Self-reported | Self-reported |
| Project Viva (United States) | Self-reported | Self-reported | Clinical records | Clinical records |
| Raine Study (Australia) | Measured | Self-reported | Measured | Last menstrual period or clinical records |
| REPRO_PL (Poland) | Measured | Self-reported | Measured | Clinical records |
| RHEA (Greece) | Measured | Self-reported | Measured | Clinical records |
| Slovak PCB study (Slovakia) | Self-reported | Self-reported | Clinical records | Clinical records |
| STEPS (Finland) | Self-reported | Self-reported | Self-reported | Self-reported |
| SWS (United Kingdom) | Measured | Measured | Measured | Last menstrual period and ultrasound |

**Table S2.** Box-Cox *t* model specifications for each maternal pre-pregnancy body mass index group

| **Maternal pre-pregnancy body mass index group** | **M-curve**  **(internal breakpoints)** | **S-curve**  **(degrees of freedom)** | **L-curve (degrees of freedom)** | **T-curve (degrees of freedom)** |
| --- | --- | --- | --- | --- |
| Underweight | 7, 19, 20, 21, 22, 30, 33, 34 | 5 | 1 | 2 |
| Normal weight | 8, 18, 20, 21, 22, 25, 30, 34 | 5 | 1 | 1 |
| Overweight | 8, 20, 21, 22, 24, 26, 36, 38 | 6 | 1 | 1 |
| Obesity Grade 1 | 19, 20, 21, 23, 25, 32, 36 | 5 | 1 | 0 |
| Obesity Grade 2 | 12, 19, 20, 23, 25, 27, 32, 36 | 6 | 1 | 1 |
| Obesity Grade 3 | 12, 14, 17, 20, 27, 31, 34, 37 | 7 | 1 | 1 |

**Table S3.** Gestational weight measurements per participating cohort and maternal pre-pregnancy body mass index group (n=679,262)

| **Cohort name, number of participants** | **Number of measurements (0 weeks and throughout pregnancy)** | **Underweight** | **Normal weight** | **Overweight** | **Obese grade 1** | **Obese grade 2** | **Obese grade 3** |
| --- | --- | --- | --- | --- | --- | --- | --- |
| ABCD, n=7,820 | 15,726 | 753 | 11,242 | 2,734 | 724 | 204 | 69 |
| ALSPAC, n= 11,344 | 42,538 | 2,085 | 31,644 | 6,459 | 1,754 | 458 | 138 |
| AOB/F, n=2,941 | 8,548 | 386 | 5,337 | 1,883 | 587 | 242 | 113 |
| Co.N.ER, n=637 | 1,262 | 114 | 948 | 164 | 31 | 4 | 1 |
| DNBC, n=42,761 | 137,251 | 5,596 | 96,052 | 25,842 | 7,398 | 1,816 | 547 |
| EDEN, n=1,875 | 5,520 | 478 | 3,605 | 977 | 332 | 104 | 24 |
| FCOU, n=3,650 | 13,208 | 1,243 | 9,511 | 1,976 | 407 | 67 | 4 |
| GASPII, n=675 | 1,346 | 110 | 1,016 | 162 | 52 | 6 | 0 |
| GECKO Drenthe, n=2,501 | 4,475 | 80 | 2,717 | 1,117 | 394 | 124 | 43 |
| Generation R, n=7,183 | 26,877 | 1,170 | 18,248 | 5,165 | 1,647 | 500 | 147 |
| Generation XXI, n=7,621 | 14,777 | 608 | 9,691 | 3,199 | 940 | 267 | 72 |
| GENESIS, n=2,218 | 4,391 | 270 | 3,298 | 653 | 140 | 30 | 0 |
| Gen3G, n=846 | 3,347 | 145 | 1,957 | 664 | 344 | 148 | 89 |
| GINIplus, n=2,329 | 4,591 | 201 | 3,536 | 669 | 143 | 40 | 2 |
| HUMIS, n=1,067 | 2,439 | 72 | 1,508 | 569 | 221 | 61 | 8 |
| INMA, n=2,561 | 8,147 | 377 | 5,709 | 1,446 | 410 | 146 | 59 |
| KOALA, n=2,812 | 5,422 | 149 | 3,817 | 1,033 | 343 | 69 | 11 |
| Krakow Cohort, n=503 | 1,001 | 121 | 793 | 76 | 11 | 0 | 0 |
| LISAplus, n=2,962 | 5,823 | 359 | 4,377 | 771 | 235 | 55 | 26 |
| LUKAS, n=417 | 832 | 18 | 494 | 204 | 76 | 35 | 5 |
| MoBa, n=88,503 | 306,022 | 9,005 | 201,294 | 67,220 | 21,137 | 5,753 | 1,613 |
| NINFEA, n=2,237 | 5,726 | 494 | 4,142 | 844 | 188 | 53 | 5 |
| PÉLAGIE, n=1,490 | 3,338 | 212 | 2,532 | 445 | 110 | 29 | 10 |
| PIAMA, n=3,459 | 6,828 | 249 | 5,232 | 1,082 | 220 | 37 | 8 |
| Piccolipiù, n=3,294 | 6,467 | 492 | 4,729 | 886 | 291 | 67 | 2 |
| PRIDE Study, n=1,513 | 3,841 | 127 | 2,828 | 667 | 183 | 34 | 2 |
| Project Viva, n=2,106 | 8,313 | 313 | 4,895 | 1,811 | 806 | 311 | 177 |
| Raine Study, n=2,791 | 8,173 | 948 | 5,745 | 959 | 372 | 109 | 40 |
| REPRO_PL, n=1,409 | 5,176 | 465 | 3,815 | 709 | 154 | 30 | 3 |
| RHEA, n=816 | 2,208 | 90 | 1,402 | 464 | 169 | 61 | 22 |
| Slovak PCB study, n=1,048 | 3,468 | 423 | 2,394 | 466 | 156 | 29 | 0 |
| STEPS, n=1,708 | 4,382 | 115 | 2,948 | 819 | 349 | 94 | 57 |
| SWS, n=3,119 | 7,799 | 116 | 4,441 | 2,145 | 737 | 273 | 87 |
| Total group, n=218,216 | 679,262 | 27,384 | 461,897 | 134,280 | 41,061 | 11,256 | 3,384 |

**Figure S2.** Sample size according to gestational age for each maternal pre-pregnancy body mass index group


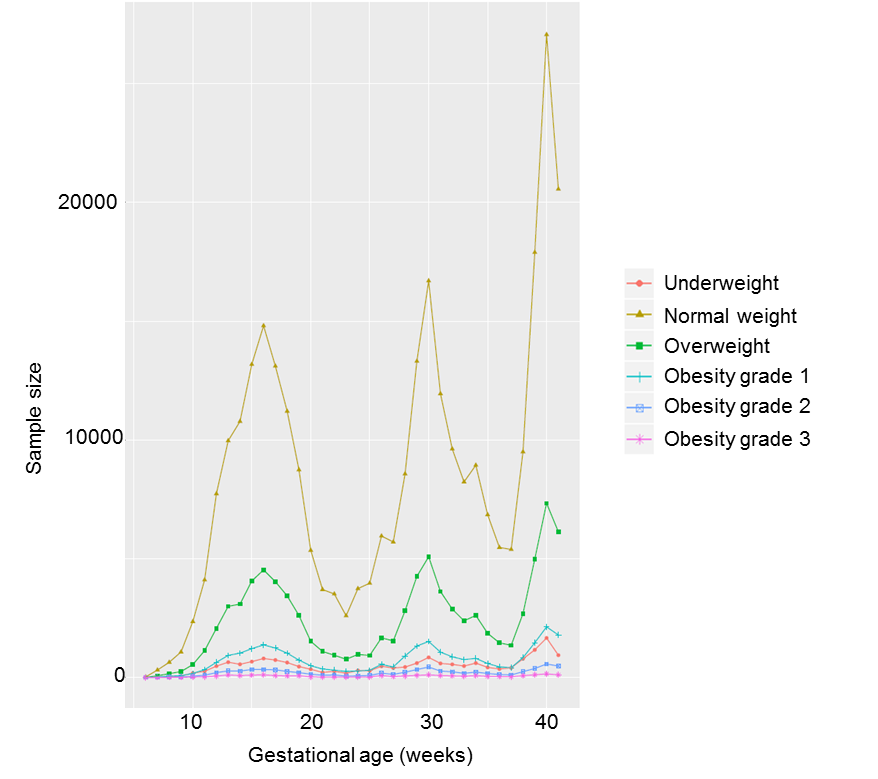


**Figure S3.** Predicted z-scores for the average weight gain according to gestational age for each maternal pre-pregnancy body mass index group


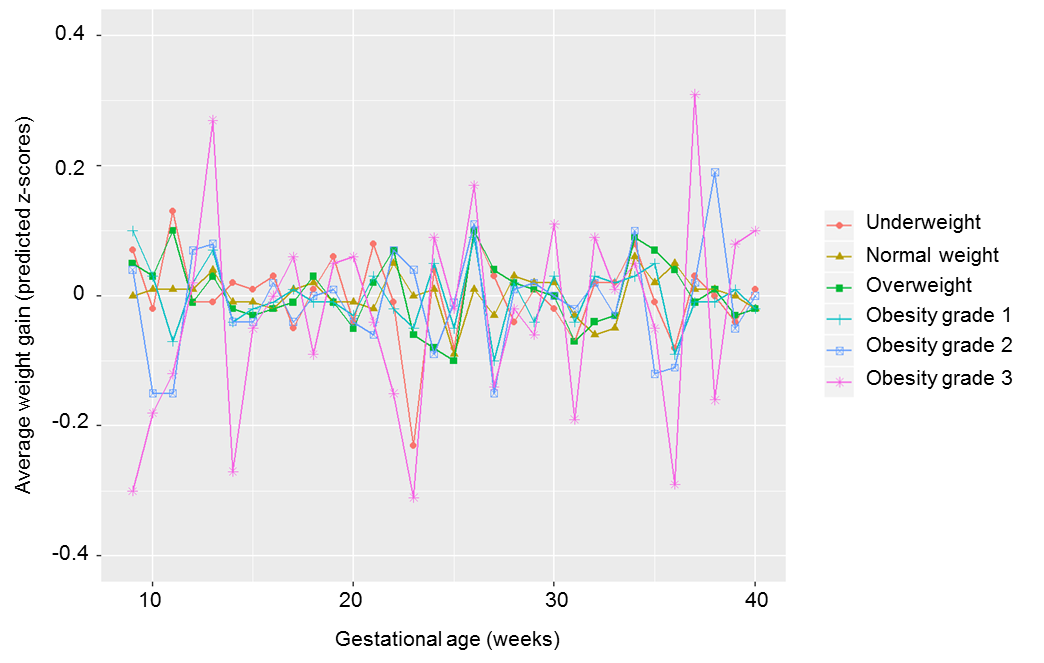


**Table S4.** Week-specific Box-Cox *t* model parameters and selected percentiles of gestational weight gain for maternal pre-pregnancy underweight

| **Gestational age (weeks)** | **Model parameters** | | | |  | **Percentiles of gestational weight gain (kg)** | | | | | | | | | | | | | | |
| --- | --- | --- | --- | --- | --- | --- | --- | --- | --- | --- | --- | --- | --- | --- | --- | --- | --- | --- | --- | --- |
|  | **Mu** | **Sigma** | **Lambda** | **Tau** |  | **P1** | **P2.3** | **P5** | **P10** | **P16** | **P20** | **P25** | **P50** | **P75** | **P80** | **P84** | **P90** | **P95** | **P97.7** | **P99** |
| 0 | 19.987 | 0.048 | 0.192 | 35.747 |  | -2.25 | -1.92 | -1.58 | -1.24 | -0.96 | -0.82 | -0.66 | -0.01 | 0.65 | 0.82 | 0.98 | 1.27 | 1.66 | 2.06 | 2.44 |
| 1 | 20.748 | 0.052 | 0.099 | 31.086 |  | -1.73 | -1.37 | -1.00 | -0.61 | -0.31 | -0.15 | 0.03 | 0.75 | 1.49 | 1.68 | 1.86 | 2.19 | 2.64 | 3.08 | 3.53 |
| 2 | 21.252 | 0.055 | 0.007 | 27.067 |  | -1.47 | -1.07 | -0.66 | -0.24 | 0.09 | 0.27 | 0.46 | 1.25 | 2.07 | 2.28 | 2.48 | 2.85 | 3.35 | 3.86 | 4.36 |
| 3 | 21.547 | 0.059 | -0.085 | 23.625 |  | -1.39 | -0.97 | -0.52 | -0.07 | 0.29 | 0.48 | 0.69 | 1.55 | 2.44 | 2.67 | 2.89 | 3.30 | 3.86 | 4.42 | 5.00 |
| 4 | 21.685 | 0.063 | -0.174 | 20.699 |  | -1.46 | -1.00 | -0.53 | -0.04 | 0.34 | 0.55 | 0.77 | 1.68 | 2.65 | 2.90 | 3.13 | 3.59 | 4.20 | 4.83 | 5.48 |
| 5 | 21.715 | 0.067 | -0.261 | 18.226 |  | -1.64 | -1.15 | -0.64 | -0.12 | 0.29 | 0.50 | 0.74 | 1.72 | 2.75 | 3.02 | 3.27 | 3.77 | 4.44 | 5.15 | 5.87 |
| 6 | 21.687 | 0.071 | -0.346 | 16.149 |  | -1.85 | -1.33 | -0.80 | -0.25 | 0.18 | 0.41 | 0.66 | 1.69 | 2.79 | 3.08 | 3.35 | 3.89 | 4.62 | 5.40 | 6.22 |
| 7 | 21.652 | 0.075 | -0.426 | 14.417 |  | -2.07 | -1.52 | -0.96 | -0.38 | 0.07 | 0.30 | 0.57 | 1.65 | 2.82 | 3.13 | 3.42 | 4.00 | 4.80 | 5.66 | 6.58 |
| 8 | 21.650 | 0.079 | -0.503 | 12.983 |  | -2.25 | -1.67 | -1.08 | -0.48 | -0.01 | 0.24 | 0.51 | 1.65 | 2.88 | 3.21 | 3.53 | 4.15 | 5.02 | 5.96 | 6.99 |
| 9 | 21.689 | 0.082 | -0.574 | 11.808 |  | -2.38 | -1.78 | -1.16 | -0.53 | -0.04 | 0.22 | 0.50 | 1.69 | 2.99 | 3.34 | 3.67 | 4.34 | 5.28 | 6.32 | 7.46 |
| 10 | 21.765 | 0.085 | -0.641 | 10.858 |  | -2.47 | -1.84 | -1.19 | -0.54 | -0.04 | 0.23 | 0.53 | 1.77 | 3.13 | 3.49 | 3.85 | 4.56 | 5.57 | 6.71 | 7.98 |
| 11 | 21.877 | 0.088 | -0.701 | 10.106 |  | -2.51 | -1.85 | -1.18 | -0.51 | 0.01 | 0.29 | 0.60 | 1.88 | 3.29 | 3.68 | 4.06 | 4.81 | 5.89 | 7.13 | 8.53 |
| 12 | 22.023 | 0.090 | -0.756 | 9.528 |  | -2.50 | -1.82 | -1.13 | -0.44 | 0.11 | 0.39 | 0.71 | 2.02 | 3.49 | 3.90 | 4.29 | 5.08 | 6.23 | 7.55 | 9.08 |
| 13 | 22.200 | 0.092 | -0.804 | 9.097 |  | -2.43 | -1.74 | -1.03 | -0.32 | 0.23 | 0.53 | 0.85 | 2.20 | 3.71 | 4.13 | 4.54 | 5.37 | 6.58 | 7.98 | 9.63 |
| 14 | 22.406 | 0.093 | -0.846 | 8.787 |  | -2.33 | -1.62 | -0.89 | -0.17 | 0.40 | 0.70 | 1.03 | 2.41 | 3.96 | 4.40 | 4.81 | 5.67 | 6.94 | 8.42 | 10.17 |
| 15 | 22.639 | 0.094 | -0.881 | 8.573 |  | -2.20 | -1.47 | -0.73 | 0.01 | 0.59 | 0.89 | 1.23 | 2.64 | 4.24 | 4.68 | 5.11 | 6.00 | 7.32 | 8.87 | 10.72 |
| 16 | 22.897 | 0.095 | -0.910 | 8.430 |  | -2.04 | -1.30 | -0.55 | 0.21 | 0.80 | 1.11 | 1.46 | 2.90 | 4.54 | 5.00 | 5.44 | 6.36 | 7.73 | 9.35 | 11.30 |
| 17 | 23.178 | 0.096 | -0.933 | 8.342 |  | -1.87 | -1.11 | -0.34 | 0.43 | 1.03 | 1.35 | 1.70 | 3.18 | 4.86 | 5.34 | 5.80 | 6.75 | 8.16 | 9.86 | 11.90 |
| 18 | 23.479 | 0.098 | -0.950 | 8.292 |  | -1.70 | -0.92 | -0.13 | 0.66 | 1.27 | 1.60 | 1.96 | 3.48 | 5.22 | 5.70 | 6.18 | 7.17 | 8.64 | 10.41 | 12.55 |
| 19 | 23.799 | 0.100 | -0.962 | 8.271 |  | -1.52 | -0.72 | 0.08 | 0.89 | 1.53 | 1.86 | 2.23 | 3.80 | 5.59 | 6.10 | 6.60 | 7.62 | 9.16 | 11.01 | 13.26 |
| 20 | 24.197 | 0.101 | -0.968 | 8.273 |  | -1.28 | -0.47 | 0.36 | 1.20 | 1.85 | 2.20 | 2.58 | 4.20 | 6.06 | 6.59 | 7.10 | 8.17 | 9.77 | 11.71 | 14.08 |
| 21 | 24.843 | 0.103 | -0.971 | 8.297 |  | -0.86 | -0.01 | 0.85 | 1.71 | 2.39 | 2.76 | 3.15 | 4.84 | 6.79 | 7.35 | 7.89 | 9.01 | 10.69 | 12.74 | 15.25 |
| 22 | 25.492 | 0.105 | -0.969 | 8.342 |  | -0.42 | 0.45 | 1.34 | 2.24 | 2.94 | 3.32 | 3.73 | 5.49 | 7.52 | 8.10 | 8.67 | 9.84 | 11.60 | 13.75 | 16.38 |

**Table S4.** Week-specific Box-Cox *t* model parameters and selected percentiles of gestational weight gain for maternal pre-pregnancy underweight (continued)

| **Gestational age (weeks)** | **Model parameters** | | | |  | **Percentiles of gestational weight gain (kg)** | | | | | | | | | | | | | | |
| --- | --- | --- | --- | --- | --- | --- | --- | --- | --- | --- | --- | --- | --- | --- | --- | --- | --- | --- | --- | --- |
|  | **Mu** | **Sigma** | **Lambda** | **Tau** |  | **P1** | **P2.3** | **P5** | **P10** | **P16** | **P20** | **P25** | **P50** | **P75** | **P80** | **P84** | **P90** | **P95** | **P97.7** | **P99** |
| 23 | 26.145 | 0.106 | -0.964 | 8.409 |  | 0.03 | 0.93 | 1.85 | 2.78 | 3.50 | 3.89 | 4.32 | 6.14 | 8.25 | 8.85 | 9.44 | 10.66 | 12.49 | 14.72 | 17.45 |
| 24 | 26.807 | 0.107 | -0.956 | 8.498 |  | 0.52 | 1.44 | 2.38 | 3.34 | 4.09 | 4.49 | 4.93 | 6.81 | 8.98 | 9.60 | 10.20 | 11.45 | 13.34 | 15.63 | 18.43 |
| 25 | 27.457 | 0.107 | -0.945 | 8.608 |  | 1.02 | 1.96 | 2.93 | 3.90 | 4.67 | 5.08 | 5.53 | 7.46 | 9.68 | 10.31 | 10.93 | 12.21 | 14.13 | 16.45 | 19.29 |
| 26 | 28.074 | 0.106 | -0.933 | 8.737 |  | 1.52 | 2.48 | 3.46 | 4.46 | 5.24 | 5.66 | 6.12 | 8.07 | 10.33 | 10.97 | 11.60 | 12.89 | 14.83 | 17.16 | 20.00 |
| 27 | 28.635 | 0.106 | -0.918 | 8.880 |  | 1.99 | 2.96 | 3.96 | 4.96 | 5.76 | 6.18 | 6.65 | 8.64 | 10.92 | 11.57 | 12.20 | 13.50 | 15.45 | 17.78 | 20.60 |
| 28 | 29.121 | 0.105 | -0.901 | 9.028 |  | 2.40 | 3.39 | 4.39 | 5.41 | 6.21 | 6.64 | 7.11 | 9.12 | 11.43 | 12.08 | 12.71 | 14.02 | 15.96 | 18.29 | 21.08 |
| 29 | 29.508 | 0.105 | -0.883 | 9.175 |  | 2.72 | 3.71 | 4.72 | 5.75 | 6.57 | 7.00 | 7.48 | 9.51 | 11.84 | 12.49 | 13.13 | 14.44 | 16.40 | 18.72 | 21.49 |
| 30 | 29.777 | 0.105 | -0.864 | 9.311 |  | 2.91 | 3.91 | 4.94 | 5.98 | 6.80 | 7.24 | 7.72 | 9.78 | 12.13 | 12.79 | 13.44 | 14.76 | 16.72 | 19.04 | 21.81 |
| 31 | 29.943 | 0.106 | -0.843 | 9.430 |  | 2.99 | 4.00 | 5.03 | 6.09 | 6.92 | 7.37 | 7.86 | 9.94 | 12.33 | 13.00 | 13.65 | 14.99 | 16.98 | 19.32 | 22.11 |
| 32 | 30.183 | 0.108 | -0.821 | 9.533 |  | 3.08 | 4.10 | 5.16 | 6.24 | 7.09 | 7.55 | 8.05 | 10.18 | 12.63 | 13.32 | 13.98 | 15.35 | 17.38 | 19.78 | 22.61 |
| 33 | 30.707 | 0.110 | -0.798 | 9.624 |  | 3.35 | 4.41 | 5.51 | 6.62 | 7.50 | 7.97 | 8.50 | 10.71 | 13.25 | 13.96 | 14.65 | 16.08 | 18.18 | 20.67 | 23.61 |
| 34 | 31.560 | 0.112 | -0.773 | 9.711 |  | 3.86 | 4.97 | 6.11 | 7.28 | 8.20 | 8.69 | 9.24 | 11.56 | 14.23 | 14.98 | 15.71 | 17.20 | 19.41 | 22.02 | 25.11 |
| 35 | 32.255 | 0.115 | -0.748 | 9.804 |  | 4.25 | 5.39 | 6.58 | 7.79 | 8.75 | 9.27 | 9.83 | 12.26 | 15.04 | 15.82 | 16.58 | 18.14 | 20.45 | 23.17 | 26.39 |
| 36 | 32.732 | 0.117 | -0.721 | 9.907 |  | 4.46 | 5.64 | 6.87 | 8.12 | 9.11 | 9.64 | 10.23 | 12.73 | 15.62 | 16.43 | 17.21 | 18.83 | 21.21 | 24.02 | 27.33 |
| 37 | 33.077 | 0.119 | -0.693 | 10.025 |  | 4.60 | 5.81 | 7.06 | 8.34 | 9.35 | 9.90 | 10.50 | 13.08 | 16.04 | 16.87 | 17.68 | 19.33 | 21.78 | 24.65 | 28.03 |
| 38 | 33.379 | 0.121 | -0.665 | 10.155 |  | 4.70 | 5.93 | 7.21 | 8.52 | 9.56 | 10.12 | 10.74 | 13.38 | 16.41 | 17.27 | 18.09 | 19.79 | 22.28 | 25.21 | 28.65 |
| 39 | 33.725 | 0.123 | -0.636 | 10.295 |  | 4.84 | 6.10 | 7.40 | 8.75 | 9.81 | 10.39 | 11.02 | 13.72 | 16.84 | 17.71 | 18.55 | 20.29 | 22.84 | 25.82 | 29.32 |
| 40 | 34.203 | 0.125 | -0.607 | 10.437 |  | 5.05 | 6.34 | 7.68 | 9.07 | 10.17 | 10.76 | 11.41 | 14.20 | 17.41 | 18.31 | 19.18 | 20.97 | 23.59 | 26.66 | 30.23 |
| 41 | 34.900 | 0.128 | -0.577 | 10.580 |  | 5.39 | 6.72 | 8.12 | 9.55 | 10.70 | 11.31 | 11.99 | 14.90 | 18.25 | 19.18 | 20.09 | 21.95 | 24.68 | 27.86 | 31.56 |
| 42 | 35.905 | 0.131 | -0.548 | 10.725 |  | 5.93 | 7.32 | 8.78 | 10.29 | 11.49 | 12.13 | 12.85 | 15.90 | 19.42 | 20.41 | 21.36 | 23.32 | 26.18 | 29.51 | 33.38 |
| 43 | 37.305 | 0.134 | -0.519 | 10.871 |  | 6.73 | 8.21 | 9.75 | 11.34 | 12.61 | 13.30 | 14.06 | 17.30 | 21.04 | 22.09 | 23.10 | 25.18 | 28.21 | 31.74 | 35.83 |
| 44 | 39.187 | 0.137 | -0.489 | 11.020 |  | 7.86 | 9.43 | 11.08 | 12.79 | 14.15 | 14.88 | 15.70 | 19.19 | 23.21 | 24.33 | 25.42 | 27.64 | 30.90 | 34.67 | 39.04 |

**Table S5.** Week-specific Box-Cox *t* model parameters and selected percentiles of gestational weight gain for maternal pre-pregnancy normal weight

| **Gestational age (weeks)** | **Model parameters** | | | |  | **Percentiles of gestational weight gain (kg)** | | | | | | | | | | | | | | |
| --- | --- | --- | --- | --- | --- | --- | --- | --- | --- | --- | --- | --- | --- | --- | --- | --- | --- | --- | --- | --- |
|  | **Mu** | **Sigma** | **Lambda** | **Tau** |  | **P1** | **P2.3** | **P5** | **P10** | **P16** | **P20** | **P25** | **P50** | **P75** | **P80** | **P84** | **P90** | **P95** | **P97.7** | **P99** |
| 0 | 19.998 | 0.049 | 0.930 | 48.097 |  | -2.33 | -1.99 | -1.63 | -1.26 | -0.98 | -0.83 | -0.66 | 0.00 | 0.66 | 0.82 | 0.98 | 1.26 | 1.63 | 2.00 | 2.35 |
| 1 | 20.199 | 0.052 | 0.794 | 35.790 |  | -2.30 | -1.93 | -1.54 | -1.15 | -0.84 | -0.68 | -0.51 | 0.20 | 0.91 | 1.09 | 1.25 | 1.57 | 1.97 | 2.37 | 2.76 |
| 2 | 20.377 | 0.054 | 0.661 | 26.745 |  | -2.30 | -1.90 | -1.48 | -1.06 | -0.74 | -0.56 | -0.38 | 0.38 | 1.14 | 1.33 | 1.51 | 1.85 | 2.29 | 2.74 | 3.18 |
| 3 | 20.536 | 0.058 | 0.533 | 20.158 |  | -2.35 | -1.90 | -1.45 | -1.00 | -0.65 | -0.47 | -0.27 | 0.54 | 1.35 | 1.56 | 1.76 | 2.13 | 2.62 | 3.12 | 3.62 |
| 4 | 20.679 | 0.061 | 0.412 | 15.390 |  | -2.43 | -1.94 | -1.45 | -0.96 | -0.59 | -0.39 | -0.18 | 0.68 | 1.56 | 1.78 | 1.99 | 2.40 | 2.95 | 3.51 | 4.09 |
| 5 | 20.811 | 0.064 | 0.302 | 11.954 |  | -2.55 | -2.01 | -1.47 | -0.94 | -0.54 | -0.33 | -0.10 | 0.81 | 1.75 | 1.99 | 2.22 | 2.67 | 3.28 | 3.92 | 4.60 |
| 6 | 20.934 | 0.067 | 0.204 | 9.486 |  | -2.71 | -2.10 | -1.50 | -0.93 | -0.50 | -0.28 | -0.03 | 0.93 | 1.94 | 2.20 | 2.45 | 2.94 | 3.62 | 4.36 | 5.16 |
| 7 | 21.051 | 0.070 | 0.121 | 7.724 |  | -2.90 | -2.22 | -1.56 | -0.93 | -0.47 | -0.23 | 0.03 | 1.05 | 2.12 | 2.41 | 2.68 | 3.22 | 3.98 | 4.83 | 5.79 |
| 8 | 21.168 | 0.073 | 0.056 | 6.479 |  | -3.12 | -2.34 | -1.62 | -0.94 | -0.44 | -0.19 | 0.09 | 1.17 | 2.30 | 2.61 | 2.90 | 3.49 | 4.35 | 5.33 | 6.47 |
| 9 | 21.286 | 0.076 | 0.009 | 5.616 |  | -3.35 | -2.48 | -1.68 | -0.94 | -0.41 | -0.14 | 0.15 | 1.29 | 2.49 | 2.81 | 3.13 | 3.77 | 4.73 | 5.85 | 7.19 |
| 10 | 21.410 | 0.079 | -0.017 | 5.039 |  | -3.55 | -2.60 | -1.72 | -0.94 | -0.37 | -0.08 | 0.22 | 1.41 | 2.67 | 3.02 | 3.36 | 4.05 | 5.09 | 6.36 | 7.90 |
| 11 | 21.544 | 0.081 | -0.021 | 4.681 |  | -3.72 | -2.68 | -1.75 | -0.91 | -0.31 | -0.01 | 0.30 | 1.54 | 2.86 | 3.23 | 3.58 | 4.32 | 5.44 | 6.83 | 8.56 |
| 12 | 21.692 | 0.083 | -0.005 | 4.501 |  | -3.82 | -2.72 | -1.73 | -0.85 | -0.23 | 0.08 | 0.41 | 1.69 | 3.06 | 3.44 | 3.81 | 4.58 | 5.76 | 7.23 | 9.10 |
| 13 | 21.858 | 0.085 | 0.028 | 4.464 |  | -3.83 | -2.69 | -1.67 | -0.76 | -0.12 | 0.20 | 0.54 | 1.86 | 3.26 | 3.65 | 4.03 | 4.82 | 6.04 | 7.56 | 9.48 |
| 14 | 22.046 | 0.086 | 0.074 | 4.531 |  | -3.77 | -2.60 | -1.56 | -0.64 | 0.02 | 0.35 | 0.70 | 2.05 | 3.48 | 3.87 | 4.26 | 5.07 | 6.30 | 7.82 | 9.74 |
| 15 | 22.259 | 0.087 | 0.122 | 4.660 |  | -3.66 | -2.48 | -1.43 | -0.48 | 0.18 | 0.52 | 0.88 | 2.26 | 3.72 | 4.13 | 4.52 | 5.33 | 6.57 | 8.09 | 9.98 |
| 16 | 22.502 | 0.089 | 0.166 | 4.820 |  | -3.52 | -2.33 | -1.26 | -0.31 | 0.38 | 0.72 | 1.08 | 2.50 | 4.00 | 4.41 | 4.81 | 5.64 | 6.88 | 8.39 | 10.26 |
| 17 | 22.779 | 0.090 | 0.201 | 4.995 |  | -3.36 | -2.16 | -1.08 | -0.10 | 0.59 | 0.95 | 1.32 | 2.78 | 4.32 | 4.74 | 5.14 | 5.98 | 7.24 | 8.76 | 10.61 |
| 18 | 23.093 | 0.093 | 0.225 | 5.171 |  | -3.20 | -1.99 | -0.88 | 0.12 | 0.84 | 1.20 | 1.58 | 3.09 | 4.68 | 5.12 | 5.54 | 6.40 | 7.68 | 9.22 | 11.08 |
| 19 | 23.455 | 0.095 | 0.238 | 5.341 |  | -3.02 | -1.79 | -0.66 | 0.37 | 1.11 | 1.49 | 1.89 | 3.46 | 5.11 | 5.56 | 5.99 | 6.89 | 8.21 | 9.78 | 11.66 |
| 20 | 23.899 | 0.097 | 0.241 | 5.507 |  | -2.79 | -1.53 | -0.37 | 0.69 | 1.46 | 1.85 | 2.26 | 3.90 | 5.62 | 6.09 | 6.55 | 7.47 | 8.84 | 10.46 | 12.39 |
| 21 | 24.385 | 0.100 | 0.237 | 5.680 |  | -2.52 | -1.24 | -0.05 | 1.04 | 1.84 | 2.24 | 2.68 | 4.38 | 6.19 | 6.68 | 7.15 | 8.12 | 9.54 | 11.21 | 13.19 |
| 22 | 24.779 | 0.102 | 0.228 | 5.870 |  | -2.29 | -1.00 | 0.21 | 1.33 | 2.14 | 2.56 | 3.01 | 4.78 | 6.65 | 7.16 | 7.65 | 8.65 | 10.11 | 11.83 | 13.86 |
| 23 | 25.557 | 0.104 | 0.214 | 6.081 |  | -1.77 | -0.45 | 0.79 | 1.95 | 2.80 | 3.24 | 3.70 | 5.56 | 7.52 | 8.06 | 8.57 | 9.62 | 11.15 | 12.93 | 15.02 |
| 24 | 26.598 | 0.106 | 0.197 | 6.320 |  | -1.02 | 0.33 | 1.61 | 2.82 | 3.70 | 4.16 | 4.65 | 6.60 | 8.67 | 9.23 | 9.77 | 10.87 | 12.47 | 14.33 | 16.49 |

**Table S5.** Week-specific Box-Cox *t* model parameters and selected percentiles of gestational weight gain for maternal pre-pregnancy normal weight (continued)

| **Gestational age (weeks)** | **Model parameters** | | | |  | **Percentiles of gestational weight gain (kg)** | | | | | | | | | | | | | | |
| --- | --- | --- | --- | --- | --- | --- | --- | --- | --- | --- | --- | --- | --- | --- | --- | --- | --- | --- | --- | --- |
|  | **Mu** | **Sigma** | **Lambda** | **Tau** |  | **P1** | **P2.3** | **P5** | **P10** | **P16** | **P20** | **P25** | **P50** | **P75** | **P80** | **P84** | **P90** | **P95** | **P97.7** | **P99** |
| 25 | 27.577 | 0.107 | 0.174 | 6.589 |  | -0.26 | 1.11 | 2.41 | 3.65 | 4.56 | 5.04 | 5.55 | 7.58 | 9.74 | 10.33 | 10.89 | 12.03 | 13.69 | 15.61 | 17.83 |
| 26 | 28.242 | 0.107 | 0.146 | 6.889 |  | 0.31 | 1.67 | 2.98 | 4.23 | 5.16 | 5.64 | 6.16 | 8.24 | 10.46 | 11.06 | 11.64 | 12.81 | 14.50 | 16.44 | 18.68 |
| 27 | 28.634 | 0.107 | 0.113 | 7.208 |  | 0.71 | 2.05 | 3.35 | 4.59 | 5.52 | 6.01 | 6.53 | 8.63 | 10.88 | 11.49 | 12.08 | 13.26 | 14.96 | 16.91 | 19.14 |
| 28 | 28.872 | 0.107 | 0.076 | 7.523 |  | 1.00 | 2.31 | 3.59 | 4.82 | 5.75 | 6.24 | 6.76 | 8.87 | 11.14 | 11.75 | 12.34 | 13.52 | 15.23 | 17.17 | 19.39 |
| 29 | 29.071 | 0.107 | 0.036 | 7.802 |  | 1.24 | 2.53 | 3.79 | 5.01 | 5.94 | 6.42 | 6.95 | 9.07 | 11.36 | 11.97 | 12.57 | 13.76 | 15.48 | 17.43 | 19.65 |
| 30 | 29.349 | 0.108 | -0.005 | 8.027 |  | 1.49 | 2.77 | 4.03 | 5.25 | 6.18 | 6.67 | 7.20 | 9.35 | 11.67 | 12.30 | 12.90 | 14.11 | 15.86 | 17.84 | 20.09 |
| 31 | 29.790 | 0.109 | -0.047 | 8.196 |  | 1.83 | 3.11 | 4.37 | 5.61 | 6.56 | 7.05 | 7.59 | 9.79 | 12.17 | 12.82 | 13.44 | 14.69 | 16.48 | 18.53 | 20.85 |
| 32 | 30.357 | 0.110 | -0.089 | 8.314 |  | 2.21 | 3.51 | 4.79 | 6.06 | 7.03 | 7.54 | 8.09 | 10.36 | 12.82 | 13.49 | 14.14 | 15.44 | 17.31 | 19.44 | 21.87 |
| 33 | 30.980 | 0.113 | -0.128 | 8.397 |  | 2.59 | 3.91 | 5.23 | 6.53 | 7.53 | 8.06 | 8.63 | 10.98 | 13.55 | 14.25 | 14.92 | 16.29 | 18.25 | 20.49 | 23.05 |
| 34 | 31.591 | 0.115 | -0.157 | 8.474 |  | 2.93 | 4.29 | 5.64 | 6.98 | 8.01 | 8.56 | 9.15 | 11.59 | 14.27 | 15.01 | 15.71 | 17.14 | 19.20 | 21.56 | 24.26 |
| 35 | 32.135 | 0.117 | -0.173 | 8.570 |  | 3.22 | 4.60 | 5.99 | 7.37 | 8.43 | 8.99 | 9.60 | 12.14 | 14.92 | 15.69 | 16.42 | 17.91 | 20.06 | 22.53 | 25.35 |
| 36 | 32.622 | 0.120 | -0.177 | 8.695 |  | 3.45 | 4.86 | 6.29 | 7.70 | 8.80 | 9.37 | 10.01 | 12.62 | 15.51 | 16.30 | 17.06 | 18.60 | 20.83 | 23.39 | 26.32 |
| 37 | 33.077 | 0.122 | -0.170 | 8.853 |  | 3.65 | 5.10 | 6.55 | 8.01 | 9.13 | 9.73 | 10.38 | 13.08 | 16.06 | 16.88 | 17.66 | 19.26 | 21.56 | 24.19 | 27.21 |
| 38 | 33.523 | 0.124 | -0.156 | 9.048 |  | 3.85 | 5.33 | 6.82 | 8.31 | 9.46 | 10.07 | 10.74 | 13.52 | 16.60 | 17.44 | 18.25 | 19.89 | 22.25 | 24.95 | 28.02 |
| 39 | 33.987 | 0.126 | -0.136 | 9.283 |  | 4.07 | 5.57 | 7.09 | 8.62 | 9.81 | 10.44 | 11.13 | 13.99 | 17.15 | 18.01 | 18.84 | 20.52 | 22.94 | 25.69 | 28.81 |
| 40 | 34.493 | 0.128 | -0.111 | 9.547 |  | 4.31 | 5.85 | 7.41 | 8.97 | 10.19 | 10.84 | 11.55 | 14.49 | 17.75 | 18.63 | 19.49 | 21.21 | 23.67 | 26.47 | 29.63 |
| 41 | 35.064 | 0.130 | -0.085 | 9.831 |  | 4.59 | 6.16 | 7.76 | 9.37 | 10.63 | 11.30 | 12.03 | 15.06 | 18.42 | 19.33 | 20.21 | 21.97 | 24.50 | 27.35 | 30.55 |
| 42 | 35.727 | 0.132 | -0.059 | 10.127 |  | 4.94 | 6.55 | 8.19 | 9.85 | 11.14 | 11.83 | 12.59 | 15.73 | 19.19 | 20.13 | 21.03 | 22.84 | 25.43 | 28.34 | 31.59 |
| 43 | 36.506 | 0.134 | -0.032 | 10.431 |  | 5.34 | 7.00 | 8.70 | 10.41 | 11.75 | 12.47 | 13.25 | 16.51 | 20.09 | 21.07 | 22.00 | 23.87 | 26.53 | 29.51 | 32.82 |
| 44 | 37.426 | 0.136 | -0.005 | 10.745 |  | 5.84 | 7.55 | 9.31 | 11.09 | 12.48 | 13.22 | 14.04 | 17.43 | 21.15 | 22.16 | 23.13 | 25.06 | 27.80 | 30.86 | 34.25 |

**Table S6.** Week-specific Box-Cox *t* model parameters and selected percentiles of gestational weight gain for maternal pre-pregnancy overweight

| **Gestational age (weeks)** | **Model parameters** | | | |  | **Percentiles of gestational weight gain (kg)** | | | | | | | | | | | | | | |
| --- | --- | --- | --- | --- | --- | --- | --- | --- | --- | --- | --- | --- | --- | --- | --- | --- | --- | --- | --- | --- |
|  | **Mu** | **Sigma** | **Lambda** | **Tau** |  | **P1** | **P2.3** | **P5** | **P10** | **P16** | **P20** | **P25** | **P50** | **P75** | **P80** | **P84** | **P90** | **P95** | **P97.7** | **P99** |
| 0 | 19.990 | 0.047 | 0.784 | 21.272 |  | -2.36 | -2.00 | -1.63 | -1.25 | -0.97 | -0.82 | -0.66 | -0.01 | 0.64 | 0.81 | 0.96 | 1.25 | 1.63 | 2.02 | 2.40 |
| 1 | 20.299 | 0.052 | 0.777 | 17.972 |  | -2.37 | -1.95 | -1.53 | -1.11 | -0.78 | -0.61 | -0.43 | 0.30 | 1.03 | 1.22 | 1.39 | 1.73 | 2.16 | 2.61 | 3.05 |
| 2 | 20.563 | 0.058 | 0.770 | 15.210 |  | -2.48 | -1.99 | -1.50 | -1.02 | -0.65 | -0.46 | -0.26 | 0.56 | 1.39 | 1.60 | 1.80 | 2.17 | 2.67 | 3.19 | 3.71 |
| 3 | 20.785 | 0.064 | 0.764 | 12.917 |  | -2.65 | -2.09 | -1.53 | -0.98 | -0.57 | -0.36 | -0.13 | 0.79 | 1.71 | 1.95 | 2.17 | 2.59 | 3.16 | 3.76 | 4.37 |
| 4 | 20.970 | 0.070 | 0.758 | 11.026 |  | -2.92 | -2.26 | -1.62 | -1.00 | -0.54 | -0.30 | -0.05 | 0.97 | 2.00 | 2.26 | 2.51 | 2.99 | 3.64 | 4.33 | 5.04 |
| 5 | 21.121 | 0.076 | 0.754 | 9.476 |  | -3.26 | -2.49 | -1.76 | -1.07 | -0.55 | -0.29 | 0.00 | 1.12 | 2.26 | 2.55 | 2.83 | 3.37 | 4.11 | 4.90 | 5.74 |
| 6 | 21.242 | 0.083 | 0.751 | 8.214 |  | -3.66 | -2.78 | -1.95 | -1.17 | -0.60 | -0.30 | 0.01 | 1.24 | 2.49 | 2.82 | 3.12 | 3.73 | 4.56 | 5.47 | 6.45 |
| 7 | 21.337 | 0.089 | 0.750 | 7.193 |  | -4.12 | -3.11 | -2.17 | -1.30 | -0.67 | -0.35 | 0.00 | 1.34 | 2.70 | 3.05 | 3.39 | 4.06 | 5.00 | 6.03 | 7.17 |
| 8 | 21.410 | 0.095 | 0.751 | 6.375 |  | -4.62 | -3.46 | -2.41 | -1.45 | -0.75 | -0.40 | -0.03 | 1.41 | 2.88 | 3.26 | 3.63 | 4.37 | 5.41 | 6.58 | 7.90 |
| 9 | 21.465 | 0.100 | 0.755 | 5.728 |  | -5.12 | -3.82 | -2.65 | -1.60 | -0.85 | -0.47 | -0.07 | 1.46 | 3.03 | 3.44 | 3.84 | 4.64 | 5.78 | 7.09 | 8.60 |
| 10 | 21.510 | 0.105 | 0.761 | 5.225 |  | -5.59 | -4.15 | -2.86 | -1.73 | -0.93 | -0.53 | -0.11 | 1.51 | 3.15 | 3.59 | 4.01 | 4.87 | 6.11 | 7.55 | 9.24 |
| 11 | 21.553 | 0.108 | 0.769 | 4.847 |  | -5.99 | -4.41 | -3.03 | -1.82 | -0.98 | -0.56 | -0.12 | 1.55 | 3.26 | 3.72 | 4.16 | 5.05 | 6.37 | 7.93 | 9.78 |
| 12 | 21.605 | 0.109 | 0.780 | 4.576 |  | -6.25 | -4.57 | -3.12 | -1.86 | -0.99 | -0.56 | -0.10 | 1.61 | 3.35 | 3.82 | 4.27 | 5.19 | 6.57 | 8.21 | 10.18 |
| 13 | 21.673 | 0.109 | 0.792 | 4.397 |  | -6.37 | -4.62 | -3.11 | -1.83 | -0.94 | -0.50 | -0.05 | 1.67 | 3.42 | 3.90 | 4.36 | 5.30 | 6.70 | 8.38 | 10.43 |
| 14 | 21.767 | 0.108 | 0.804 | 4.293 |  | -6.37 | -4.58 | -3.05 | -1.74 | -0.85 | -0.41 | 0.04 | 1.77 | 3.52 | 3.99 | 4.45 | 5.39 | 6.80 | 8.51 | 10.59 |
| 15 | 21.894 | 0.108 | 0.815 | 4.251 |  | -6.29 | -4.48 | -2.94 | -1.62 | -0.73 | -0.29 | 0.17 | 1.89 | 3.64 | 4.12 | 4.58 | 5.52 | 6.94 | 8.65 | 10.73 |
| 16 | 22.064 | 0.108 | 0.824 | 4.258 |  | -6.18 | -4.35 | -2.80 | -1.47 | -0.57 | -0.13 | 0.33 | 2.06 | 3.82 | 4.30 | 4.76 | 5.71 | 7.13 | 8.84 | 10.93 |
| 17 | 22.286 | 0.108 | 0.830 | 4.308 |  | -6.05 | -4.21 | -2.64 | -1.30 | -0.39 | 0.06 | 0.53 | 2.29 | 4.07 | 4.55 | 5.02 | 5.98 | 7.41 | 9.14 | 11.23 |
| 18 | 22.568 | 0.110 | 0.831 | 4.393 |  | -5.91 | -4.05 | -2.46 | -1.10 | -0.17 | 0.29 | 0.76 | 2.57 | 4.40 | 4.89 | 5.37 | 6.35 | 7.80 | 9.55 | 11.66 |
| 19 | 22.919 | 0.112 | 0.829 | 4.510 |  | -5.78 | -3.90 | -2.27 | -0.88 | 0.08 | 0.55 | 1.05 | 2.92 | 4.82 | 5.33 | 5.82 | 6.83 | 8.33 | 10.12 | 12.26 |
| 20 | 23.347 | 0.116 | 0.823 | 4.655 |  | -5.62 | -3.71 | -2.05 | -0.61 | 0.38 | 0.88 | 1.39 | 3.35 | 5.33 | 5.87 | 6.38 | 7.43 | 8.98 | 10.82 | 13.02 |
| 21 | 23.695 | 0.119 | 0.813 | 4.828 |  | -5.54 | -3.61 | -1.91 | -0.43 | 0.60 | 1.11 | 1.65 | 3.69 | 5.77 | 6.33 | 6.87 | 7.96 | 9.57 | 11.46 | 13.70 |
| 22 | 23.717 | 0.124 | 0.801 | 5.026 |  | -5.65 | -3.73 | -2.02 | -0.52 | 0.53 | 1.06 | 1.61 | 3.72 | 5.86 | 6.44 | 6.99 | 8.11 | 9.75 | 11.67 | 13.93 |
| 23 | 24.733 | 0.127 | 0.786 | 5.250 |  | -5.13 | -3.15 | -1.37 | 0.21 | 1.33 | 1.89 | 2.47 | 4.73 | 7.04 | 7.65 | 8.24 | 9.44 | 11.18 | 13.20 | 15.56 |
| 24 | 26.130 | 0.131 | 0.769 | 5.499 |  | -4.35 | -2.28 | -0.41 | 1.26 | 2.46 | 3.06 | 3.69 | 6.13 | 8.63 | 9.29 | 9.93 | 11.22 | 13.08 | 15.23 | 17.72 |

**Table S6.** Week-specific Box-Cox *t* model parameters and selected percentiles of gestational weight gain for maternal pre-pregnancy overweight (continued)

| **Gestational age (weeks)** | **Model parameters** | | | |  | **Percentiles of gestational weight gain (kg)** | | | | | | | | | | | | | | |
| --- | --- | --- | --- | --- | --- | --- | --- | --- | --- | --- | --- | --- | --- | --- | --- | --- | --- | --- | --- | --- |
|  | **Mu** | **Sigma** | **Lambda** | **Tau** |  | **P1** | **P2.3** | **P5** | **P10** | **P16** | **P20** | **P25** | **P50** | **P75** | **P80** | **P84** | **P90** | **P95** | **P97.7** | **P99** |
| 25 | 26.949 | 0.133 | 0.751 | 5.770 |  | -3.84 | -1.75 | 0.15 | 1.88 | 3.11 | 3.73 | 4.39 | 6.95 | 9.57 | 10.26 | 10.93 | 12.27 | 14.20 | 16.42 | 18.97 |
| 26 | 27.339 | 0.135 | 0.732 | 6.061 |  | -3.50 | -1.44 | 0.45 | 2.18 | 3.43 | 4.06 | 4.73 | 7.34 | 10.02 | 10.73 | 11.41 | 12.78 | 14.73 | 16.97 | 19.51 |
| 27 | 27.662 | 0.135 | 0.712 | 6.370 |  | -3.13 | -1.12 | 0.75 | 2.47 | 3.71 | 4.35 | 5.02 | 7.66 | 10.37 | 11.10 | 11.78 | 13.16 | 15.12 | 17.34 | 19.86 |
| 28 | 27.994 | 0.135 | 0.691 | 6.691 |  | -2.72 | -0.76 | 1.08 | 2.78 | 4.03 | 4.66 | 5.34 | 7.99 | 10.73 | 11.46 | 12.15 | 13.53 | 15.49 | 17.69 | 20.17 |
| 29 | 28.344 | 0.135 | 0.671 | 7.017 |  | -2.32 | -0.40 | 1.41 | 3.10 | 4.35 | 4.98 | 5.67 | 8.34 | 11.11 | 11.84 | 12.54 | 13.93 | 15.89 | 18.09 | 20.55 |
| 30 | 28.719 | 0.136 | 0.650 | 7.342 |  | -1.97 | -0.08 | 1.72 | 3.41 | 4.67 | 5.31 | 6.00 | 8.72 | 11.53 | 12.28 | 12.98 | 14.39 | 16.38 | 18.59 | 21.04 |
| 31 | 29.125 | 0.138 | 0.628 | 7.661 |  | -1.69 | 0.20 | 2.00 | 3.71 | 4.98 | 5.64 | 6.34 | 9.12 | 12.01 | 12.77 | 13.50 | 14.94 | 16.96 | 19.21 | 21.70 |
| 32 | 29.569 | 0.140 | 0.607 | 7.973 |  | -1.47 | 0.42 | 2.25 | 4.00 | 5.30 | 5.97 | 6.69 | 9.57 | 12.56 | 13.35 | 14.10 | 15.59 | 17.68 | 19.99 | 22.54 |
| 33 | 30.059 | 0.144 | 0.586 | 8.283 |  | -1.30 | 0.62 | 2.48 | 4.28 | 5.62 | 6.32 | 7.07 | 10.06 | 13.18 | 14.01 | 14.79 | 16.34 | 18.52 | 20.92 | 23.56 |
| 34 | 30.603 | 0.149 | 0.567 | 8.596 |  | -1.13 | 0.82 | 2.73 | 4.58 | 5.97 | 6.69 | 7.48 | 10.60 | 13.87 | 14.74 | 15.56 | 17.19 | 19.47 | 21.98 | 24.72 |
| 35 | 31.207 | 0.153 | 0.549 | 8.919 |  | -0.93 | 1.06 | 3.02 | 4.93 | 6.37 | 7.12 | 7.94 | 11.21 | 14.64 | 15.55 | 16.41 | 18.11 | 20.50 | 23.12 | 25.97 |
| 36 | 31.878 | 0.157 | 0.534 | 9.256 |  | -0.67 | 1.36 | 3.37 | 5.34 | 6.84 | 7.62 | 8.47 | 11.88 | 15.47 | 16.42 | 17.32 | 19.11 | 21.60 | 24.33 | 27.29 |
| 37 | 32.591 | 0.160 | 0.520 | 9.610 |  | -0.35 | 1.72 | 3.78 | 5.80 | 7.35 | 8.16 | 9.04 | 12.59 | 16.34 | 17.33 | 18.27 | 20.14 | 22.73 | 25.56 | 28.63 |
| 38 | 33.191 | 0.163 | 0.507 | 9.981 |  | -0.07 | 2.03 | 4.12 | 6.20 | 7.78 | 8.62 | 9.52 | 13.19 | 17.07 | 18.10 | 19.07 | 21.00 | 23.68 | 26.60 | 29.75 |
| 39 | 33.561 | 0.165 | 0.495 | 10.371 |  | 0.13 | 2.23 | 4.34 | 6.43 | 8.04 | 8.89 | 9.81 | 13.56 | 17.53 | 18.58 | 19.58 | 21.55 | 24.28 | 27.25 | 30.45 |
| 40 | 33.863 | 0.166 | 0.482 | 10.782 |  | 0.34 | 2.43 | 4.54 | 6.65 | 8.27 | 9.13 | 10.06 | 13.86 | 17.90 | 18.96 | 19.97 | 21.97 | 24.74 | 27.74 | 30.96 |
| 41 | 34.329 | 0.167 | 0.469 | 11.214 |  | 0.68 | 2.77 | 4.89 | 7.01 | 8.65 | 9.52 | 10.47 | 14.33 | 18.44 | 19.52 | 20.55 | 22.58 | 25.39 | 28.43 | 31.69 |
| 42 | 35.191 | 0.167 | 0.456 | 11.664 |  | 1.25 | 3.37 | 5.52 | 7.68 | 9.36 | 10.25 | 11.22 | 15.19 | 19.42 | 20.54 | 21.60 | 23.69 | 26.57 | 29.68 | 33.01 |
| 43 | 36.682 | 0.168 | 0.443 | 12.132 |  | 2.21 | 4.38 | 6.61 | 8.85 | 10.60 | 11.52 | 12.53 | 16.68 | 21.11 | 22.28 | 23.39 | 25.57 | 28.59 | 31.83 | 35.29 |
| 44 | 39.034 | 0.169 | 0.429 | 12.618 |  | 3.68 | 5.97 | 8.31 | 10.69 | 12.54 | 13.53 | 14.61 | 19.03 | 23.77 | 25.02 | 26.21 | 28.54 | 31.76 | 35.22 | 38.89 |

**Table S7.** Week-specific Box-Cox *t* model parameters and selected percentiles of gestational weight gain for maternal pre-pregnancy obesity grade 1

| **Gestational age (weeks)** | **Model parameters** | | | |  | **Percentiles of gestational weight gain (kg)** | | | | | | | | | | | | | | |
| --- | --- | --- | --- | --- | --- | --- | --- | --- | --- | --- | --- | --- | --- | --- | --- | --- | --- | --- | --- | --- |
|  | **Mu** | **Sigma** | **Lambda** | **Tau** |  | **P1** | **P2.3** | **P5** | **P10** | **P16** | **P20** | **P25** | **P50** | **P75** | **P80** | **P84** | **P90** | **P95** | **P97.7** | **P99** |
| 0 | 20.005 | 0.046 | 0.948 | 11.258 |  | -2.46 | -2.04 | -1.63 | -1.24 | -0.95 | -0.79 | -0.63 | 0.00 | 0.64 | 0.81 | 0.96 | 1.25 | 1.65 | 2.06 | 2.49 |
| 1 | 20.416 | 0.051 | 0.955 | 10.798 |  | -2.43 | -1.94 | -1.46 | -1.01 | -0.67 | -0.50 | -0.31 | 0.42 | 1.15 | 1.33 | 1.51 | 1.85 | 2.30 | 2.78 | 3.28 |
| 2 | 20.735 | 0.058 | 0.961 | 10.358 |  | -2.53 | -1.96 | -1.41 | -0.89 | -0.51 | -0.31 | -0.10 | 0.73 | 1.57 | 1.78 | 1.98 | 2.37 | 2.89 | 3.44 | 4.02 |
| 3 | 20.973 | 0.064 | 0.967 | 9.941 |  | -2.75 | -2.10 | -1.47 | -0.88 | -0.44 | -0.21 | 0.03 | 0.97 | 1.92 | 2.16 | 2.39 | 2.83 | 3.43 | 4.06 | 4.72 |
| 4 | 21.138 | 0.072 | 0.973 | 9.547 |  | -3.08 | -2.33 | -1.62 | -0.95 | -0.45 | -0.20 | 0.07 | 1.14 | 2.20 | 2.48 | 2.73 | 3.23 | 3.91 | 4.62 | 5.38 |
| 5 | 21.242 | 0.080 | 0.978 | 9.177 |  | -3.50 | -2.65 | -1.85 | -1.09 | -0.54 | -0.25 | 0.05 | 1.24 | 2.43 | 2.74 | 3.02 | 3.58 | 4.34 | 5.15 | 6.01 |
| 6 | 21.292 | 0.088 | 0.983 | 8.832 |  | -4.01 | -3.05 | -2.15 | -1.30 | -0.68 | -0.37 | -0.03 | 1.29 | 2.61 | 2.95 | 3.27 | 3.89 | 4.74 | 5.65 | 6.62 |
| 7 | 21.301 | 0.097 | 0.988 | 8.511 |  | -4.57 | -3.50 | -2.50 | -1.56 | -0.87 | -0.52 | -0.15 | 1.30 | 2.75 | 3.13 | 3.48 | 4.16 | 5.11 | 6.11 | 7.19 |
| 8 | 21.276 | 0.105 | 0.992 | 8.215 |  | -5.16 | -3.98 | -2.87 | -1.84 | -1.09 | -0.71 | -0.30 | 1.28 | 2.86 | 3.26 | 3.65 | 4.40 | 5.43 | 6.54 | 7.73 |
| 9 | 21.228 | 0.114 | 0.996 | 7.943 |  | -5.77 | -4.47 | -3.26 | -2.14 | -1.33 | -0.92 | -0.48 | 1.23 | 2.93 | 3.37 | 3.79 | 4.60 | 5.72 | 6.93 | 8.23 |
| 10 | 21.166 | 0.121 | 0.999 | 7.696 |  | -6.34 | -4.94 | -3.64 | -2.44 | -1.57 | -1.12 | -0.65 | 1.17 | 2.98 | 3.46 | 3.90 | 4.77 | 5.97 | 7.27 | 8.68 |
| 11 | 21.101 | 0.128 | 1.001 | 7.474 |  | -6.85 | -5.35 | -3.97 | -2.70 | -1.78 | -1.31 | -0.81 | 1.10 | 3.01 | 3.51 | 3.98 | 4.90 | 6.17 | 7.55 | 9.06 |
| 12 | 21.042 | 0.133 | 1.003 | 7.276 |  | -7.25 | -5.68 | -4.23 | -2.90 | -1.95 | -1.46 | -0.94 | 1.04 | 3.03 | 3.54 | 4.03 | 4.98 | 6.31 | 7.76 | 9.34 |
| 13 | 20.998 | 0.136 | 1.003 | 7.102 |  | -7.53 | -5.90 | -4.40 | -3.04 | -2.06 | -1.56 | -1.03 | 1.00 | 3.03 | 3.56 | 4.05 | 5.03 | 6.40 | 7.89 | 9.53 |
| 14 | 20.980 | 0.138 | 1.002 | 6.951 |  | -7.68 | -6.02 | -4.50 | -3.11 | -2.11 | -1.61 | -1.07 | 0.98 | 3.04 | 3.57 | 4.07 | 5.07 | 6.46 | 7.98 | 9.66 |
| 15 | 20.998 | 0.138 | 0.999 | 6.821 |  | -7.75 | -6.06 | -4.52 | -3.12 | -2.12 | -1.61 | -1.07 | 1.00 | 3.07 | 3.60 | 4.11 | 5.12 | 6.52 | 8.07 | 9.77 |
| 16 | 21.060 | 0.139 | 0.994 | 6.712 |  | -7.77 | -6.06 | -4.50 | -3.09 | -2.07 | -1.56 | -1.02 | 1.06 | 3.14 | 3.69 | 4.20 | 5.21 | 6.63 | 8.20 | 9.93 |
| 17 | 21.177 | 0.140 | 0.987 | 6.621 |  | -7.78 | -6.04 | -4.46 | -3.02 | -2.00 | -1.48 | -0.93 | 1.18 | 3.29 | 3.84 | 4.36 | 5.39 | 6.84 | 8.44 | 10.21 |
| 18 | 21.359 | 0.141 | 0.977 | 6.549 |  | -7.78 | -6.01 | -4.39 | -2.93 | -1.88 | -1.35 | -0.79 | 1.36 | 3.51 | 4.08 | 4.61 | 5.67 | 7.15 | 8.79 | 10.62 |
| 19 | 21.615 | 0.144 | 0.966 | 6.493 |  | -7.78 | -5.96 | -4.30 | -2.79 | -1.71 | -1.17 | -0.60 | 1.62 | 3.83 | 4.41 | 4.96 | 6.05 | 7.59 | 9.29 | 11.18 |
| 20 | 21.945 | 0.146 | 0.952 | 6.453 |  | -7.74 | -5.87 | -4.16 | -2.60 | -1.49 | -0.93 | -0.34 | 1.95 | 4.24 | 4.84 | 5.41 | 6.54 | 8.14 | 9.91 | 11.88 |
| 21 | 22.287 | 0.149 | 0.937 | 6.428 |  | -7.72 | -5.79 | -4.02 | -2.42 | -1.27 | -0.69 | -0.08 | 2.29 | 4.67 | 5.29 | 5.88 | 7.06 | 8.72 | 10.57 | 12.62 |
| 22 | 22.560 | 0.152 | 0.921 | 6.417 |  | -7.71 | -5.74 | -3.93 | -2.28 | -1.10 | -0.51 | 0.12 | 2.56 | 5.02 | 5.66 | 6.27 | 7.49 | 9.21 | 11.13 | 13.27 |
| 23 | 23.035 | 0.154 | 0.903 | 6.418 |  | -7.57 | -5.54 | -3.68 | -1.98 | -0.76 | -0.14 | 0.51 | 3.04 | 5.59 | 6.26 | 6.90 | 8.16 | 9.95 | 11.96 | 14.19 |
| 24 | 23.933 | 0.157 | 0.884 | 6.433 |  | -7.17 | -5.06 | -3.12 | -1.34 | -0.06 | 0.59 | 1.28 | 3.93 | 6.63 | 7.34 | 8.01 | 9.35 | 11.24 | 13.36 | 15.73 |

**Table S7**. Week-specific Box-Cox *t* model parameters and selected percentiles of gestational weight gain for maternal pre-pregnancy obesity grade 1 (continued)

| **Gestational age (weeks)** | **Model parameters** | | | |  | **Percentiles of gestational weight gain (kg)** | | | | | | | | | | | | | | |
| --- | --- | --- | --- | --- | --- | --- | --- | --- | --- | --- | --- | --- | --- | --- | --- | --- | --- | --- | --- | --- |
|  | **Mu** | **Sigma** | **Lambda** | **Tau** |  | **P1** | **P2.3** | **P5** | **P10** | **P16** | **P20** | **P25** | **P50** | **P75** | **P80** | **P84** | **P90** | **P95** | **P97.7** | **P99** |
| 25 | 24.916 | 0.158 | 0.864 | 6.458 |  | -6.69 | -4.49 | -2.47 | -0.61 | 0.73 | 1.40 | 2.13 | 4.92 | 7.75 | 8.50 | 9.21 | 10.62 | 12.62 | 14.86 | 17.37 |
| 26 | 25.599 | 0.159 | 0.843 | 6.495 |  | -6.31 | -4.07 | -2.00 | -0.10 | 1.28 | 1.98 | 2.72 | 5.60 | 8.53 | 9.31 | 10.04 | 11.51 | 13.58 | 15.91 | 18.51 |
| 27 | 25.985 | 0.160 | 0.822 | 6.541 |  | -6.04 | -3.80 | -1.71 | 0.21 | 1.60 | 2.30 | 3.06 | 5.99 | 8.97 | 9.76 | 10.51 | 12.01 | 14.12 | 16.50 | 19.16 |
| 28 | 26.172 | 0.160 | 0.800 | 6.597 |  | -5.88 | -3.65 | -1.57 | 0.36 | 1.75 | 2.46 | 3.22 | 6.17 | 9.19 | 9.99 | 10.75 | 12.26 | 14.40 | 16.81 | 19.50 |
| 29 | 26.259 | 0.160 | 0.779 | 6.661 |  | -5.77 | -3.56 | -1.49 | 0.43 | 1.82 | 2.54 | 3.29 | 6.26 | 9.30 | 10.10 | 10.87 | 12.40 | 14.56 | 16.99 | 19.71 |
| 30 | 26.344 | 0.162 | 0.756 | 6.732 |  | -5.70 | -3.51 | -1.45 | 0.47 | 1.87 | 2.59 | 3.35 | 6.34 | 9.42 | 10.23 | 11.01 | 12.56 | 14.75 | 17.21 | 19.96 |
| 31 | 26.527 | 0.164 | 0.734 | 6.811 |  | -5.63 | -3.43 | -1.36 | 0.57 | 1.99 | 2.71 | 3.49 | 6.53 | 9.66 | 10.49 | 11.28 | 12.86 | 15.10 | 17.62 | 20.43 |
| 32 | 26.905 | 0.167 | 0.712 | 6.896 |  | -5.52 | -3.30 | -1.20 | 0.78 | 2.23 | 2.97 | 3.77 | 6.90 | 10.15 | 11.01 | 11.83 | 13.47 | 15.79 | 18.39 | 21.31 |
| 33 | 27.538 | 0.171 | 0.690 | 6.986 |  | -5.36 | -3.08 | -0.91 | 1.14 | 2.65 | 3.42 | 4.25 | 7.54 | 10.95 | 11.85 | 12.72 | 14.44 | 16.89 | 19.64 | 22.71 |
| 34 | 28.327 | 0.176 | 0.668 | 7.082 |  | -5.16 | -2.81 | -0.55 | 1.59 | 3.17 | 3.99 | 4.86 | 8.33 | 11.94 | 12.90 | 13.81 | 15.65 | 18.25 | 21.17 | 24.44 |
| 35 | 29.134 | 0.181 | 0.646 | 7.184 |  | -4.96 | -2.53 | -0.18 | 2.05 | 3.70 | 4.56 | 5.48 | 9.13 | 12.96 | 13.98 | 14.95 | 16.90 | 19.66 | 22.76 | 26.24 |
| 36 | 29.823 | 0.185 | 0.625 | 7.290 |  | -4.79 | -2.30 | 0.11 | 2.42 | 4.15 | 5.04 | 6.00 | 9.82 | 13.84 | 14.92 | 15.94 | 17.99 | 20.90 | 24.17 | 27.84 |
| 37 | 30.294 | 0.189 | 0.605 | 7.401 |  | -4.68 | -2.16 | 0.30 | 2.66 | 4.43 | 5.35 | 6.34 | 10.29 | 14.47 | 15.58 | 16.65 | 18.78 | 21.81 | 25.22 | 29.04 |
| 38 | 30.611 | 0.192 | 0.584 | 7.516 |  | -4.60 | -2.07 | 0.41 | 2.81 | 4.61 | 5.55 | 6.56 | 10.61 | 14.90 | 16.05 | 17.15 | 19.34 | 22.47 | 25.98 | 29.91 |
| 39 | 30.874 | 0.195 | 0.564 | 7.634 |  | -4.49 | -1.96 | 0.53 | 2.95 | 4.77 | 5.72 | 6.75 | 10.87 | 15.26 | 16.44 | 17.56 | 19.81 | 23.01 | 26.61 | 30.64 |
| 40 | 31.187 | 0.196 | 0.543 | 7.756 |  | -4.30 | -1.78 | 0.72 | 3.15 | 4.99 | 5.95 | 6.99 | 11.19 | 15.66 | 16.86 | 18.00 | 20.30 | 23.57 | 27.25 | 31.37 |
| 41 | 31.650 | 0.197 | 0.523 | 7.880 |  | -4.00 | -1.47 | 1.04 | 3.49 | 5.35 | 6.33 | 7.38 | 11.65 | 16.21 | 17.44 | 18.61 | 20.96 | 24.31 | 28.07 | 32.28 |
| 42 | 32.367 | 0.198 | 0.503 | 8.006 |  | -3.55 | -1.00 | 1.54 | 4.03 | 5.92 | 6.92 | 8.00 | 12.37 | 17.05 | 18.32 | 19.52 | 21.94 | 25.38 | 29.25 | 33.58 |
| 43 | 33.438 | 0.199 | 0.482 | 8.135 |  | -2.92 | -0.32 | 2.27 | 4.83 | 6.78 | 7.80 | 8.91 | 13.44 | 18.30 | 19.61 | 20.87 | 23.38 | 26.96 | 30.99 | 35.49 |
| 44 | 34.966 | 0.200 | 0.462 | 8.265 |  | -2.05 | 0.63 | 3.31 | 5.97 | 8.00 | 9.07 | 10.23 | 14.97 | 20.08 | 21.46 | 22.77 | 25.42 | 29.19 | 33.42 | 38.17 |

**Table S8.** Week-specific Box-Cox *t* model parameters and selected percentiles of gestational weight gain for maternal pre-pregnancy obesity grade 2

| **Gestational age (weeks)** | **Model parameters** | | | |  | **Percentiles of gestational weight gain (kg)** | | | | | | | | | | | | | | |
| --- | --- | --- | --- | --- | --- | --- | --- | --- | --- | --- | --- | --- | --- | --- | --- | --- | --- | --- | --- | --- |
|  | **Mu** | **Sigma** | **Lambda** | **Tau** |  | **P1** | **P2.3** | **P5** | **P10** | **P16** | **P20** | **P25** | **P50** | **P75** | **P80** | **P84** | **P90** | **P95** | **P97.7** | **P99** |
| 0 | 20.014 | 0.048 | 1.042 | 20.073 |  | -2.42 | -2.03 | -1.65 | -1.26 | -0.97 | -0.81 | -0.65 | 0.01 | 0.67 | 0.84 | 0.99 | 1.29 | 1.67 | 2.05 | 2.44 |
| 1 | 19.999 | 0.055 | 1.041 | 18.409 |  | -2.81 | -2.36 | -1.91 | -1.46 | -1.12 | -0.95 | -0.76 | 0.00 | 0.75 | 0.94 | 1.12 | 1.46 | 1.90 | 2.34 | 2.79 |
| 2 | 20.028 | 0.063 | 1.040 | 16.893 |  | -3.20 | -2.68 | -2.16 | -1.65 | -1.26 | -1.06 | -0.84 | 0.03 | 0.89 | 1.11 | 1.31 | 1.70 | 2.21 | 2.72 | 3.24 |
| 3 | 20.092 | 0.071 | 1.039 | 15.521 |  | -3.64 | -3.03 | -2.42 | -1.83 | -1.38 | -1.15 | -0.90 | 0.09 | 1.08 | 1.33 | 1.56 | 2.01 | 2.59 | 3.19 | 3.79 |
| 4 | 20.180 | 0.081 | 1.037 | 14.286 |  | -4.11 | -3.40 | -2.70 | -2.02 | -1.50 | -1.24 | -0.95 | 0.18 | 1.31 | 1.59 | 1.86 | 2.37 | 3.04 | 3.73 | 4.43 |
| 5 | 20.284 | 0.091 | 1.035 | 13.183 |  | -4.63 | -3.81 | -3.00 | -2.22 | -1.63 | -1.33 | -1.00 | 0.28 | 1.57 | 1.89 | 2.19 | 2.77 | 3.55 | 4.34 | 5.16 |
| 6 | 20.393 | 0.102 | 1.033 | 12.201 |  | -5.21 | -4.26 | -3.33 | -2.44 | -1.77 | -1.43 | -1.06 | 0.39 | 1.84 | 2.21 | 2.55 | 3.21 | 4.09 | 5.01 | 5.94 |
| 7 | 20.498 | 0.114 | 1.031 | 11.335 |  | -5.85 | -4.75 | -3.69 | -2.68 | -1.93 | -1.55 | -1.13 | 0.50 | 2.12 | 2.53 | 2.92 | 3.66 | 4.66 | 5.70 | 6.78 |
| 8 | 20.589 | 0.125 | 1.028 | 10.575 |  | -6.51 | -5.27 | -4.08 | -2.95 | -2.11 | -1.68 | -1.22 | 0.59 | 2.39 | 2.85 | 3.28 | 4.11 | 5.23 | 6.40 | 7.62 |
| 9 | 20.656 | 0.137 | 1.024 | 9.915 |  | -7.19 | -5.80 | -4.48 | -3.23 | -2.30 | -1.83 | -1.32 | 0.66 | 2.63 | 3.13 | 3.61 | 4.52 | 5.76 | 7.07 | 8.44 |
| 10 | 20.691 | 0.147 | 1.020 | 9.347 |  | -7.84 | -6.31 | -4.86 | -3.50 | -2.50 | -1.99 | -1.44 | 0.69 | 2.82 | 3.36 | 3.87 | 4.87 | 6.21 | 7.65 | 9.15 |
| 11 | 20.682 | 0.154 | 1.015 | 8.866 |  | -8.37 | -6.73 | -5.19 | -3.74 | -2.68 | -2.14 | -1.56 | 0.68 | 2.92 | 3.50 | 4.04 | 5.09 | 6.53 | 8.06 | 9.68 |
| 12 | 20.622 | 0.159 | 1.009 | 8.465 |  | -8.73 | -7.03 | -5.42 | -3.93 | -2.84 | -2.28 | -1.68 | 0.62 | 2.93 | 3.52 | 4.07 | 5.16 | 6.65 | 8.25 | 9.95 |
| 13 | 20.506 | 0.160 | 1.003 | 8.137 |  | -8.93 | -7.19 | -5.57 | -4.06 | -2.96 | -2.40 | -1.80 | 0.51 | 2.82 | 3.41 | 3.97 | 5.07 | 6.58 | 8.21 | 9.95 |
| 14 | 20.366 | 0.159 | 0.996 | 7.875 |  | -9.00 | -7.26 | -5.65 | -4.15 | -3.06 | -2.51 | -1.92 | 0.37 | 2.65 | 3.24 | 3.80 | 4.89 | 6.39 | 8.02 | 9.77 |
| 15 | 20.235 | 0.157 | 0.987 | 7.670 |  | -9.02 | -7.30 | -5.70 | -4.22 | -3.14 | -2.60 | -2.01 | 0.24 | 2.49 | 3.07 | 3.62 | 4.70 | 6.19 | 7.82 | 9.57 |
| 16 | 20.152 | 0.157 | 0.978 | 7.516 |  | -9.05 | -7.34 | -5.74 | -4.27 | -3.20 | -2.66 | -2.08 | 0.15 | 2.39 | 2.97 | 3.52 | 4.60 | 6.09 | 7.72 | 9.48 |
| 17 | 20.153 | 0.158 | 0.966 | 7.408 |  | -9.11 | -7.38 | -5.77 | -4.29 | -3.22 | -2.67 | -2.09 | 0.15 | 2.41 | 2.99 | 3.54 | 4.63 | 6.14 | 7.79 | 9.59 |
| 18 | 20.273 | 0.159 | 0.954 | 7.344 |  | -9.14 | -7.39 | -5.75 | -4.25 | -3.16 | -2.60 | -2.01 | 0.27 | 2.57 | 3.17 | 3.73 | 4.85 | 6.39 | 8.08 | 9.93 |
| 19 | 20.549 | 0.162 | 0.940 | 7.318 |  | -9.12 | -7.32 | -5.65 | -4.11 | -2.98 | -2.41 | -1.80 | 0.55 | 2.92 | 3.54 | 4.12 | 5.27 | 6.87 | 8.63 | 10.54 |
| 20 | 20.934 | 0.165 | 0.925 | 7.329 |  | -9.07 | -7.22 | -5.49 | -3.89 | -2.73 | -2.14 | -1.51 | 0.93 | 3.40 | 4.04 | 4.65 | 5.85 | 7.52 | 9.35 | 11.35 |
| 21 | 21.168 | 0.170 | 0.909 | 7.371 |  | -9.12 | -7.24 | -5.46 | -3.82 | -2.62 | -2.01 | -1.36 | 1.17 | 3.73 | 4.39 | 5.02 | 6.27 | 8.01 | 9.91 | 11.99 |
| 22 | 21.409 | 0.174 | 0.892 | 7.443 |  | -9.19 | -7.26 | -5.44 | -3.75 | -2.52 | -1.89 | -1.21 | 1.41 | 4.07 | 4.76 | 5.42 | 6.71 | 8.52 | 10.50 | 12.67 |
| 23 | 21.941 | 0.179 | 0.874 | 7.542 |  | -9.11 | -7.12 | -5.23 | -3.47 | -2.18 | -1.52 | -0.81 | 1.94 | 4.74 | 5.47 | 6.16 | 7.53 | 9.43 | 11.52 | 13.80 |
| 24 | 22.895 | 0.183 | 0.857 | 7.666 |  | -8.80 | -6.71 | -4.73 | -2.87 | -1.50 | -0.79 | -0.04 | 2.90 | 5.89 | 6.68 | 7.42 | 8.88 | 10.92 | 13.15 | 15.59 |

**Table S8.** Week-specific Box-Cox *t* model parameters and selected percentiles of gestational weight gain for maternal pre-pregnancy obesity grade 2 (continued)

| **Gestational age (weeks)** | **Model parameters** | | | |  | **Percentiles of gestational weight gain (kg)** | | | | | | | | | | | | | | |
| --- | --- | --- | --- | --- | --- | --- | --- | --- | --- | --- | --- | --- | --- | --- | --- | --- | --- | --- | --- | --- |
|  | **Mu** | **Sigma** | **Lambda** | **Tau** |  | **P1** | **P2.3** | **P5** | **P10** | **P16** | **P20** | **P25** | **P50** | **P75** | **P80** | **P84** | **P90** | **P95** | **P97.7** | **P99** |
| 25 | 23.796 | 0.187 | 0.839 | 7.813 |  | -8.45 | -6.29 | -4.23 | -2.28 | -0.84 | -0.10 | 0.69 | 3.80 | 6.97 | 7.80 | 8.58 | 10.14 | 12.29 | 14.66 | 17.24 |
| 26 | 24.174 | 0.189 | 0.821 | 7.982 |  | -8.29 | -6.12 | -4.03 | -2.05 | -0.58 | 0.18 | 0.99 | 4.17 | 7.44 | 8.29 | 9.10 | 10.70 | 12.92 | 15.35 | 18.00 |
| 27 | 24.206 | 0.190 | 0.803 | 8.168 |  | -8.23 | -6.08 | -4.01 | -2.04 | -0.57 | 0.19 | 1.00 | 4.21 | 7.50 | 8.36 | 9.18 | 10.79 | 13.03 | 15.47 | 18.13 |
| 28 | 24.183 | 0.191 | 0.786 | 8.370 |  | -8.14 | -6.03 | -3.99 | -2.04 | -0.58 | 0.17 | 0.98 | 4.18 | 7.48 | 8.34 | 9.16 | 10.77 | 13.01 | 15.45 | 18.10 |
| 29 | 24.207 | 0.191 | 0.768 | 8.584 |  | -8.01 | -5.94 | -3.93 | -1.99 | -0.55 | 0.20 | 1.01 | 4.21 | 7.51 | 8.37 | 9.19 | 10.80 | 13.04 | 15.48 | 18.12 |
| 30 | 24.333 | 0.191 | 0.751 | 8.809 |  | -7.86 | -5.82 | -3.82 | -1.89 | -0.44 | 0.31 | 1.12 | 4.33 | 7.66 | 8.53 | 9.36 | 10.99 | 13.24 | 15.69 | 18.35 |
| 31 | 24.616 | 0.193 | 0.734 | 9.043 |  | -7.69 | -5.65 | -3.65 | -1.70 | -0.24 | 0.52 | 1.34 | 4.62 | 8.01 | 8.90 | 9.74 | 11.40 | 13.70 | 16.20 | 18.90 |
| 32 | 25.112 | 0.196 | 0.717 | 9.283 |  | -7.52 | -5.45 | -3.40 | -1.41 | 0.09 | 0.88 | 1.72 | 5.11 | 8.64 | 9.56 | 10.43 | 12.16 | 14.55 | 17.14 | 19.92 |
| 33 | 25.840 | 0.201 | 0.701 | 9.530 |  | -7.32 | -5.19 | -3.07 | -1.00 | 0.57 | 1.39 | 2.28 | 5.84 | 9.56 | 10.53 | 11.46 | 13.28 | 15.80 | 18.53 | 21.46 |
| 34 | 26.682 | 0.206 | 0.685 | 9.783 |  | -7.10 | -4.90 | -2.70 | -0.54 | 1.11 | 1.98 | 2.91 | 6.68 | 10.63 | 11.66 | 12.64 | 14.58 | 17.25 | 20.15 | 23.26 |
| 35 | 27.485 | 0.212 | 0.671 | 10.042 |  | -6.90 | -4.63 | -2.35 | -0.10 | 1.63 | 2.53 | 3.52 | 7.48 | 11.65 | 12.75 | 13.79 | 15.83 | 18.66 | 21.72 | 25.00 |
| 36 | 28.094 | 0.216 | 0.657 | 10.307 |  | -6.78 | -4.46 | -2.12 | 0.21 | 2.00 | 2.94 | 3.96 | 8.09 | 12.45 | 13.60 | 14.68 | 16.83 | 19.78 | 22.98 | 26.41 |
| 37 | 28.406 | 0.220 | 0.644 | 10.579 |  | -6.74 | -4.40 | -2.03 | 0.33 | 2.16 | 3.12 | 4.16 | 8.41 | 12.89 | 14.07 | 15.19 | 17.40 | 20.44 | 23.74 | 27.26 |
| 38 | 28.518 | 0.223 | 0.631 | 10.857 |  | -6.75 | -4.42 | -2.04 | 0.34 | 2.18 | 3.15 | 4.21 | 8.52 | 13.08 | 14.29 | 15.43 | 17.68 | 20.78 | 24.12 | 27.70 |
| 39 | 28.576 | 0.226 | 0.619 | 11.141 |  | -6.76 | -4.44 | -2.07 | 0.32 | 2.17 | 3.15 | 4.22 | 8.58 | 13.21 | 14.43 | 15.58 | 17.87 | 21.01 | 24.40 | 28.02 |
| 40 | 28.726 | 0.228 | 0.608 | 11.431 |  | -6.74 | -4.42 | -2.04 | 0.36 | 2.23 | 3.22 | 4.30 | 8.73 | 13.44 | 14.68 | 15.86 | 18.18 | 21.39 | 24.83 | 28.51 |
| 41 | 29.115 | 0.231 | 0.596 | 11.729 |  | -6.60 | -4.27 | -1.86 | 0.57 | 2.48 | 3.48 | 4.59 | 9.12 | 13.95 | 15.23 | 16.43 | 18.82 | 22.10 | 25.64 | 29.40 |
| 42 | 29.890 | 0.233 | 0.584 | 12.035 |  | -6.25 | -3.88 | -1.42 | 1.08 | 3.03 | 4.07 | 5.21 | 9.89 | 14.89 | 16.22 | 17.47 | 19.94 | 23.35 | 27.01 | 30.90 |
| 43 | 31.196 | 0.235 | 0.573 | 12.349 |  | -5.63 | -3.18 | -0.63 | 1.97 | 4.01 | 5.10 | 6.29 | 11.20 | 16.45 | 17.85 | 19.16 | 21.76 | 25.34 | 29.18 | 33.27 |
| 44 | 33.180 | 0.236 | 0.561 | 12.672 |  | -4.69 | -2.11 | 0.59 | 3.34 | 5.51 | 6.67 | 7.94 | 13.18 | 18.81 | 20.30 | 21.72 | 24.50 | 28.33 | 32.45 | 36.81 |

**Table S9.** Week-specific Box-Cox *t* model parameters and selected percentiles of gestational weight gain for maternal pre-pregnancy obesity grade 3

| **Gestational age (weeks)** | **Model parameters** | | | |  | **Percentiles of gestational weight gain (kg)** | | | | | | | | | | | | | | |
| --- | --- | --- | --- | --- | --- | --- | --- | --- | --- | --- | --- | --- | --- | --- | --- | --- | --- | --- | --- | --- |
|  | **Mu** | **Sigma** | **Lambda** | **Tau** |  | **P1** | **P2.3** | **P5** | **P10** | **P16** | **P20** | **P25** | **P50** | **P75** | **P80** | **P84** | **P90** | **P95** | **P97.7** | **P99** |
| 0 | 19.983 | 0.047 | 0.896 | 17.468 |  | -2.39 | -2.01 | -1.63 | -1.25 | -0.97 | -0.82 | -0.66 | -0.02 | 0.63 | 0.79 | 0.94 | 1.23 | 1.61 | 1.99 | 2.38 |
| 1 | 19.057 | 0.053 | 0.915 | 15.994 |  | -3.53 | -3.11 | -2.69 | -2.28 | -1.97 | -1.81 | -1.64 | -0.94 | -0.25 | -0.07 | 0.09 | 0.41 | 0.82 | 1.24 | 1.67 |
| 2 | 18.548 | 0.060 | 0.935 | 14.656 |  | -4.33 | -3.86 | -3.39 | -2.93 | -2.59 | -2.41 | -2.22 | -1.45 | -0.69 | -0.49 | -0.31 | 0.04 | 0.50 | 0.97 | 1.45 |
| 3 | 18.384 | 0.067 | 0.955 | 13.452 |  | -4.87 | -4.33 | -3.80 | -3.28 | -2.89 | -2.69 | -2.47 | -1.62 | -0.76 | -0.54 | -0.33 | 0.06 | 0.58 | 1.12 | 1.67 |
| 4 | 18.494 | 0.076 | 0.974 | 12.376 |  | -5.24 | -4.61 | -3.99 | -3.40 | -2.96 | -2.73 | -2.48 | -1.51 | -0.53 | -0.28 | -0.05 | 0.39 | 0.99 | 1.61 | 2.24 |
| 5 | 18.806 | 0.085 | 0.993 | 11.424 |  | -5.50 | -4.77 | -4.05 | -3.37 | -2.85 | -2.59 | -2.31 | -1.19 | -0.08 | 0.20 | 0.47 | 0.98 | 1.66 | 2.38 | 3.12 |
| 6 | 19.249 | 0.095 | 1.012 | 10.587 |  | -5.74 | -4.87 | -4.04 | -3.24 | -2.65 | -2.35 | -2.02 | -0.75 | 0.52 | 0.84 | 1.15 | 1.74 | 2.53 | 3.36 | 4.22 |
| 7 | 19.751 | 0.105 | 1.030 | 9.859 |  | -6.00 | -4.98 | -4.01 | -3.09 | -2.42 | -2.07 | -1.70 | -0.25 | 1.20 | 1.57 | 1.91 | 2.58 | 3.49 | 4.45 | 5.45 |
| 8 | 20.242 | 0.115 | 1.049 | 9.234 |  | -6.33 | -5.15 | -4.03 | -2.98 | -2.21 | -1.81 | -1.39 | 0.24 | 1.87 | 2.29 | 2.67 | 3.43 | 4.47 | 5.56 | 6.71 |
| 9 | 20.649 | 0.125 | 1.066 | 8.702 |  | -6.77 | -5.41 | -4.13 | -2.95 | -2.08 | -1.64 | -1.17 | 0.65 | 2.46 | 2.92 | 3.36 | 4.20 | 5.36 | 6.59 | 7.90 |
| 10 | 20.902 | 0.135 | 1.083 | 8.254 |  | -7.37 | -5.82 | -4.39 | -3.07 | -2.11 | -1.62 | -1.10 | 0.90 | 2.89 | 3.40 | 3.88 | 4.81 | 6.09 | 7.45 | 8.91 |
| 11 | 20.928 | 0.146 | 1.098 | 7.882 |  | -8.15 | -6.43 | -4.84 | -3.39 | -2.34 | -1.81 | -1.24 | 0.93 | 3.08 | 3.63 | 4.15 | 5.16 | 6.55 | 8.04 | 9.64 |
| 12 | 20.657 | 0.157 | 1.112 | 7.581 |  | -9.09 | -7.22 | -5.50 | -3.94 | -2.82 | -2.25 | -1.65 | 0.66 | 2.94 | 3.52 | 4.07 | 5.15 | 6.63 | 8.23 | 9.94 |
| 13 | 20.093 | 0.165 | 1.125 | 7.346 |  | -10.02 | -8.07 | -6.28 | -4.65 | -3.49 | -2.90 | -2.28 | 0.09 | 2.43 | 3.04 | 3.60 | 4.71 | 6.23 | 7.89 | 9.67 |
| 14 | 19.541 | 0.171 | 1.135 | 7.169 |  | -10.70 | -8.72 | -6.89 | -5.24 | -4.06 | -3.47 | -2.84 | -0.46 | 1.90 | 2.50 | 3.07 | 4.18 | 5.72 | 7.39 | 9.20 |
| 15 | 19.295 | 0.173 | 1.143 | 7.043 |  | -11.02 | -9.01 | -7.17 | -5.51 | -4.32 | -3.73 | -3.10 | -0.70 | 1.65 | 2.26 | 2.83 | 3.95 | 5.49 | 7.17 | 8.99 |
| 16 | 19.286 | 0.174 | 1.149 | 6.963 |  | -11.13 | -9.10 | -7.23 | -5.55 | -4.35 | -3.75 | -3.12 | -0.71 | 1.66 | 2.27 | 2.84 | 3.97 | 5.52 | 7.21 | 9.05 |
| 17 | 19.357 | 0.177 | 1.152 | 6.920 |  | -11.27 | -9.20 | -7.29 | -5.58 | -4.35 | -3.74 | -3.10 | -0.64 | 1.77 | 2.39 | 2.98 | 4.12 | 5.71 | 7.43 | 9.31 |
| 18 | 19.392 | 0.182 | 1.153 | 6.911 |  | -11.55 | -9.43 | -7.47 | -5.70 | -4.44 | -3.80 | -3.14 | -0.60 | 1.88 | 2.52 | 3.12 | 4.31 | 5.94 | 7.72 | 9.65 |
| 19 | 19.452 | 0.187 | 1.150 | 6.932 |  | -11.80 | -9.63 | -7.62 | -5.79 | -4.49 | -3.84 | -3.16 | -0.54 | 2.02 | 2.68 | 3.30 | 4.52 | 6.20 | 8.04 | 10.02 |
| 20 | 19.639 | 0.190 | 1.144 | 6.982 |  | -11.85 | -9.64 | -7.59 | -5.73 | -4.40 | -3.73 | -3.03 | -0.35 | 2.27 | 2.95 | 3.58 | 4.83 | 6.55 | 8.43 | 10.46 |
| 21 | 20.029 | 0.190 | 1.135 | 7.060 |  | -11.62 | -9.39 | -7.32 | -5.43 | -4.08 | -3.41 | -2.69 | 0.03 | 2.71 | 3.40 | 4.05 | 5.32 | 7.08 | 8.98 | 11.05 |
| 22 | 20.576 | 0.189 | 1.123 | 7.164 |  | -11.24 | -8.98 | -6.89 | -4.97 | -3.61 | -2.92 | -2.19 | 0.58 | 3.31 | 4.02 | 4.68 | 5.97 | 7.76 | 9.70 | 11.80 |
| 23 | 21.206 | 0.188 | 1.108 | 7.290 |  | -10.83 | -8.54 | -6.41 | -4.47 | -3.08 | -2.37 | -1.63 | 1.21 | 4.01 | 4.74 | 5.41 | 6.75 | 8.58 | 10.57 | 12.72 |

**Table S9.** Week-specific Box-Cox *t* model parameters and selected percentiles of gestational weight gain for maternal pre-pregnancy obesity grade 3 (continued)

| **Gestational age (weeks)** | **Model parameters** | | | |  | **Percentiles of gestational weight gain (kg)** | | | | | | | | | | | | | | |
| --- | --- | --- | --- | --- | --- | --- | --- | --- | --- | --- | --- | --- | --- | --- | --- | --- | --- | --- | --- | --- |
|  | **Mu** | **Sigma** | **Lambda** | **Tau** |  | **P1** | **P2.3** | **P5** | **P10** | **P16** | **P20** | **P25** | **P50** | **P75** | **P80** | **P84** | **P90** | **P95** | **P97.7** | **P99** |
| 24 | 21.847 | 0.188 | 1.090 | 7.440 |  | -10.48 | -8.15 | -5.99 | -4.00 | -2.57 | -1.85 | -1.08 | 1.85 | 4.75 | 5.50 | 6.20 | 7.57 | 9.47 | 11.52 | 13.74 |
| 25 | 22.424 | 0.191 | 1.069 | 7.611 |  | -10.25 | -7.89 | -5.67 | -3.63 | -2.16 | -1.41 | -0.62 | 2.43 | 5.45 | 6.22 | 6.95 | 8.39 | 10.36 | 12.49 | 14.78 |
| 26 | 22.864 | 0.195 | 1.046 | 7.804 |  | -10.13 | -7.73 | -5.48 | -3.39 | -1.87 | -1.10 | -0.28 | 2.87 | 6.00 | 6.81 | 7.57 | 9.06 | 11.10 | 13.31 | 15.68 |
| 27 | 23.092 | 0.197 | 1.020 | 8.019 |  | -10.04 | -7.65 | -5.38 | -3.27 | -1.74 | -0.96 | -0.12 | 3.09 | 6.31 | 7.14 | 7.91 | 9.44 | 11.54 | 13.80 | 16.22 |
| 28 | 23.072 | 0.200 | 0.991 | 8.253 |  | -10.05 | -7.69 | -5.45 | -3.35 | -1.81 | -1.02 | -0.18 | 3.07 | 6.34 | 7.18 | 7.97 | 9.52 | 11.65 | 13.94 | 16.40 |
| 29 | 22.915 | 0.206 | 0.959 | 8.503 |  | -10.22 | -7.90 | -5.67 | -3.57 | -2.03 | -1.23 | -0.39 | 2.92 | 6.24 | 7.10 | 7.91 | 9.50 | 11.67 | 14.01 | 16.51 |
| 30 | 22.770 | 0.213 | 0.924 | 8.768 |  | -10.47 | -8.18 | -5.96 | -3.84 | -2.28 | -1.47 | -0.61 | 2.77 | 6.19 | 7.08 | 7.91 | 9.55 | 11.79 | 14.20 | 16.77 |
| 31 | 22.783 | 0.220 | 0.886 | 9.046 |  | -10.64 | -8.36 | -6.14 | -4.00 | -2.41 | -1.58 | -0.70 | 2.79 | 6.33 | 7.25 | 8.12 | 9.82 | 12.15 | 14.66 | 17.33 |
| 32 | 23.074 | 0.227 | 0.845 | 9.337 |  | -10.66 | -8.38 | -6.13 | -3.95 | -2.31 | -1.46 | -0.55 | 3.08 | 6.79 | 7.76 | 8.67 | 10.46 | 12.91 | 15.55 | 18.37 |
| 33 | 23.637 | 0.235 | 0.802 | 9.641 |  | -10.61 | -8.30 | -6.00 | -3.75 | -2.04 | -1.15 | -0.19 | 3.64 | 7.60 | 8.63 | 9.60 | 11.52 | 14.15 | 16.99 | 20.01 |
| 34 | 24.436 | 0.245 | 0.756 | 9.960 |  | -10.50 | -8.14 | -5.77 | -3.41 | -1.62 | -0.68 | 0.34 | 4.44 | 8.71 | 9.83 | 10.89 | 12.97 | 15.84 | 18.93 | 22.23 |
| 35 | 25.394 | 0.253 | 0.707 | 10.295 |  | -10.22 | -7.82 | -5.37 | -2.93 | -1.04 | -0.05 | 1.03 | 5.40 | 10.00 | 11.21 | 12.36 | 14.62 | 17.73 | 21.10 | 24.70 |
| 36 | 26.257 | 0.259 | 0.656 | 10.648 |  | -9.86 | -7.45 | -4.96 | -2.45 | -0.50 | 0.53 | 1.66 | 6.26 | 11.16 | 12.45 | 13.68 | 16.11 | 19.46 | 23.10 | 26.99 |
| 37 | 26.730 | 0.265 | 0.604 | 11.020 |  | -9.58 | -7.20 | -4.73 | -2.20 | -0.22 | 0.83 | 1.98 | 6.73 | 11.84 | 13.20 | 14.49 | 17.04 | 20.58 | 24.43 | 28.56 |
| 38 | 26.642 | 0.267 | 0.550 | 11.412 |  | -9.36 | -7.09 | -4.69 | -2.23 | -0.28 | 0.75 | 1.90 | 6.64 | 11.80 | 13.18 | 14.49 | 17.10 | 20.72 | 24.66 | 28.90 |
| 39 | 26.333 | 0.263 | 0.496 | 11.827 |  | -8.98 | -6.84 | -4.58 | -2.23 | -0.37 | 0.63 | 1.73 | 6.33 | 11.38 | 12.74 | 14.03 | 16.59 | 20.17 | 24.08 | 28.29 |
| 40 | 26.267 | 0.254 | 0.441 | 12.265 |  | -8.26 | -6.25 | -4.12 | -1.90 | -0.14 | 0.81 | 1.86 | 6.27 | 11.13 | 12.44 | 13.68 | 16.17 | 19.64 | 23.43 | 27.52 |
| 41 | 26.909 | 0.241 | 0.387 | 12.723 |  | -7.12 | -5.19 | -3.14 | -1.01 | 0.70 | 1.62 | 2.63 | 6.91 | 11.65 | 12.93 | 14.15 | 16.58 | 19.97 | 23.69 | 27.70 |
| 42 | 28.724 | 0.228 | 0.332 | 13.201 |  | -5.34 | -3.41 | -1.37 | 0.77 | 2.48 | 3.40 | 4.42 | 8.72 | 13.51 | 14.80 | 16.03 | 18.49 | 21.93 | 25.69 | 29.76 |
| 43 | 32.177 | 0.215 | 0.277 | 13.697 |  | -2.63 | -0.61 | 1.54 | 3.78 | 5.58 | 6.55 | 7.63 | 12.18 | 17.24 | 18.61 | 19.92 | 22.52 | 26.17 | 30.16 | 34.47 |
| 44 | 37.733 | 0.203 | 0.222 | 14.212 |  | 1.41 | 3.63 | 5.99 | 8.46 | 10.45 | 11.52 | 12.71 | 17.73 | 23.34 | 24.86 | 26.31 | 29.20 | 33.24 | 37.65 | 42.42 |

**Table S10.** Local institutional ethical review boards per cohort

| **Cohort name (country)** | **Local institutional ethical review boards** |
| --- | --- |
| ABCD (The Netherlands) | Central Committee on Research Involving Human Subjects in The Netherlands, the medical ethics review committees of the participating hospitals and the Registration Committee of the Municipality of Amsterdam |
| ALSPAC (United Kingdom) | ALSPAC Ethics and Law Committee and Local Research Ethics Committees |
| AOB/F (Canada) | Child Health Research Office and the Conjoint Health Research Ethics Board of the Faculties of Medicine, Nursing, and Kinesiology, University of Calgary, and the Affiliated Teaching Institutions (Ethics ID 20821 and 22821) |
| Co.N.ER (Italy) | Ethics Committee of the S. Orsola-Malpighi Teaching Hospital of Bologna (Italy) (052/2004/U/Tess) |
| DNBC (Denmark) | The Scientific Ethic Committee in Denmark, the Danish Data Protection Agency, and the DNBC Steering Committee |
| EDEN (France) | Ethics Committee of the Bicêtre Hospital |
| FCOU (Ukraine) | Institutional Review Boards at the University of Illinois at Chicago and the Ukrainian Institute for Pediatrics, Obstetrics, and Gynecology |
| GASPII (Italy) | Ethical Committee of the Università Cattolica del Sacro Cuore, Rome |
| GECKO Drenthe (The Netherlands) | Medical Ethics Committee of the University Medical Center Groningen (UMCG) |
| Generation R (The Netherlands) | Medical Ethical Committee of the Erasmus Medical Center, Rotterdam |
| Generation XXI (Portugal) | Ethics Committee of Hospital de S. João |
| GENESIS (Greece) | Ethical Committee of Harokopio University of Athens and all municipalities invited to participate in the study |
| Gen3G (Canada) | CHUS Ethics Review Board for Studies with Humans |
| GINIplus (Germany) | Bavarian General Medical Council, University of Leipzig, Medical Council of North-Rhine-Westphalia |
| HUMIS (Norway) | Regional Ethics Committee for Medical Research in Norway (reference S-02122) and Norwegian Data Inspectorate |
| INMA (Spain) | The Municipal Institute of Sanitary Assistance of Barcelona, La Fe University Hospital of Valencia, The Donostia Hospital, and Ib-salut |
| KOALA (The Netherlands) | Medical ethics committee of the Maastricht University/University Hospital of Maastricht |
| Krakow Cohort (Poland) | The Bioethical Committee of Jagiellonian University |
| LISAplus (Germany) | Bavarian General Medical Council, University of Leipzig, Medical Council of North-Rhine-Westphalia |
| LUKAS (Finland) | Research Ethics Committee, Hospital District of Northern Savo, Kuopio, Finland |
| MoBa (Norway) | Norwegian Data Inspectorate and the Regional Ethics Committee for Medical Research |
| NINFEA (Italy) | Ethical Committee of the San Giovanni Battista Hospital and CTO/CRF/Maria Adelaide Hospital of Turin (approval N.0048362 and following amendments) |
| PÉLAGIE (France) | French Consulting Committee for the Treatment of Information in Medical Research (no. 09.485) and the French National Commission for the Confidentiality of Computerised Data (no. 909347) |
| PIAMA (The Netherlands) | Rotterdam, MEC (Medisch Ethische Commisie Erasmus Universiteit Rotterdam/Academische Ziekenhuizen Rotterdam), Groningen, MEC (Medisch Ethische Commisie Academisch ziekenhuis Groningen) and Utrecht/Bilthoven, MEC-TNO (Medisch Ethische Commisie -Toegepast Natuurwetenschappelijk Onderzoek) |
| Piccolipiù (Italy) | Ethics committees of the Local Health Unit Roma E (management centre), of the Istituto Superiore di Sanità (National Institute of Public Health) and of each local centre |
| PRIDE Study (The Netherlands) | Regional Committee on Research involving Human Subjects |
| Project Viva (United States) | Institutional Review Board of Harvard Pilgrim Health Care |
| Raine Study (Australia) | The University of Western Australia Human Research Ethics Committee |
| REPRO_PL (Poland) | Ethical Committee of the Nofer Institute of Occupational Medicine, Łódź, Po­land (Decision No. 7/2007) |
| RHEA (Greece) | Ethical Committee of the University Hospital, Scientific Council, Heraklion, Crete, Greece |
| Slovak PCB study (Slovakia) | Institutional review boards at the University of California, Davis, and the Slovak Medical University |
| STEPS (Finland) | The Ministry of Social Affairs and Health and the Ethics Committee of the Hospital District of Southwest Finland |
| SWS (United Kingdom) | Southampton and South West Hampshire Local Research Ethics Committee (06/Q1702/104) |
